# Toll-like Receptors in Immuno-Metabolic Regulation of Emotion and Memory

**DOI:** 10.3390/cells14120933

**Published:** 2025-06-19

**Authors:** Carla Crespo-Quiles, Teresa Femenía

**Affiliations:** 1 Instituto de Neurociencias, Universidad Miguel Hernández de Elche (UMH) and Consejo Superior de Investigaciones Científicas (CSIC), 03550 San Juan de Alicante, Spain; c.crespo@umh.es; 2 Redes de Investigación Cooperativa Orientada a Resultados en Salud, Red de Investigación en Atención Primaria de Adicciones, Instituto de Salud Carlos III, Ministerio de Ciencia e Innovación (MICINN) and Fondo Europeo de Desarrollo Regional (FEDER), 28029 Madrid, Spain

**Keywords:** Toll-like receptors, emotion, cognition, psychiatry, metabolism, neuroplasticity

## Abstract

Toll-like receptors (TLRs) comprise an evolutionarily conserved family of pattern recognition receptors that detect microbial-associated molecular patterns and endogenous danger signals to orchestrate innate immune responses. While traditionally positioned at the frontline of host defense, accumulating evidence suggests that TLRs are at the nexus of immuno-metabolic regulation and central nervous system (CNS) homeostasis. They regulate a wide range of immune and non-immune functions, such as cytokine and chemokine signaling, and play key roles in modulating synaptic plasticity, neurogenesis, and neuronal survival. However, alterations in TLR signaling can drive a sustained pro-inflammatory state, mitochondrial dysfunction, and oxidative stress, which are highly associated with the disruption of emotional and cognitive functions and the pathogenesis of psychiatric disorders. In this review, we integrate findings from molecular to organismal levels to illustrate the diverse roles of TLRs in regulating emotion, cognition, metabolic balance, and gut–brain interactions. We also explore emerging molecular targets with the potential to guide the development of more effective therapeutic interventions.

## 1. Introduction

The immune system is a complex network of cells, tissues, organs, and signaling molecules that maintain homeostasis by combating external insults, such as infections and toxins, as well as internal threats, including cancer and tissue damage [1,2,3]. Central to this defense mechanism are pattern recognition receptors (PRRs), in particular Toll-like receptors (TLRs), which recognize exogenous molecules from organisms known as microorganism-associated molecular patterns (MAMPs) and endogenous danger signals called damage-associated molecular patterns (DAMPs) to activate inflammatory cascades. The discovery of TLRs represents a landmark in immunology, bridging the fields of developmental biology and innate immunity. In the 1980s, Christiane Nüsslein-Volhard and Eric Wieschaus identified the *toll* gene as essential for embryonic polarity in *Drosophila* [4,5], a finding that earned them the Nobel Prize in Physiology or Medicine in 1995. Building on this, Jules A. Hoffmann demonstrated in 1996 that *toll* also mediates antifungal immune responses in flies [6]. Subsequently, Bruce A. Beutler showed in 1998 that TLR4 in mammals is responsible for sensing lipopolysaccharide (LPS), a key component of Gram-negative bacteria, revealing evolutionary conservation in pathogen recognition [7]. The contributions of Hoffmann and Beutler were recognized with the Nobel Prize in Physiology or Medicine in 2011. Together, these milestones revolutionized our understanding of how ancient developmental pathways evolved to detect pathogens and launch protective innate immune responses [8,9], serving as the first line of defense against pathogens and tissue injury. They are non-specific, meaning they recognize a broad range of threats, and their activation is rapid and immediate. TLRs are evolutionarily conserved, and their activation leads to the production of inflammatory cytokines and other immune responses [10,11]. Recent studies have underscored the critical influence of TLR signaling not only in immune responses but also in the modulation of metabolic regulation, emotional behavior, and cognitive functions, processes increasingly implicated in the pathophysiology of psychiatric and neurodegenerative disorders.

Inflammation, a hallmark of the immune response, is initiated by the rapid activation of key immune cells, including dendritic cells, macrophages, natural killer (NK) cells, and granulocytes. These cells secrete cytokines and chemokines that mediate vasodilation, cell recruitment, and increased vascular permeability, setting the stage for both acute pathogen clearance and tissue repair [12]. However, when inflammation becomes chronic, as seen in obesity, arthritis, and gastrointestinal disorders, this persistent inflammatory state disrupts normal homeostasis and is associated with alterations in mood and cognition [3,13,14]. For example, chronic neuroinflammation is associated with reduced hippocampal volume, impaired neuronal plasticity, and deficits in learning and memory, all of which are common features of many psychiatric disorders, including depression and anxiety [15,16]

Recent evidence suggests that TLRs are at the nexus of immuno-metabolic crosstalk. Metabolic alterations, often associated with chronic inflammation, contribute to the pathogenesis of mood and anxiety disorders as well as cognitive decline [13,17,18,19,20]. Therapeutic interventions such as antidepressants and physical exercise have been shown to counteract these effects by enhancing neurogenesis and synaptic plasticity, ultimately promoting cognitive recovery [21,22,23,24]. In particular, TLR signaling in peripheral tissues, such as adipose tissue and the gut, plays a crucial role in regulating systemic metabolic homeostasis. Dysbiosis or changes in the gut microbiota can activate TLRs and trigger inflammatory responses that affect neurotransmission and neuroplasticity in the brain. Conversely, targeting the gut microbiota with probiotics or dietary modifications has been shown to modulate TLR-driven inflammation, thereby improving neuronal plasticity and emotional outcomes [25,26]

This review focuses on the role of TLRs in mediating immune and non-immune responses that impact emotional, cognitive, and metabolic functions, drawing on evidence from clinical studies, rodent animal models with translational relevance for psychiatry, and cellular models. By integrating findings from molecular, cellular, and in vivo studies, we aim to elucidate the mechanisms by which TLRs contribute to anxiety and mood disorders in psychiatry. Understanding these mechanisms is crucial for the development of targeted therapies that effectively mitigate the risk of chronic inflammation, ultimately enhancing outcomes in neuropsychiatric and metabolic disorders.

## 2. Toll-like Receptors and Their Associated Immune Mechanisms

TLRs are type I transmembrane glycoproteins characterized by an extracellular domain containing leucine-rich repeat (LRR) regions, through which they recognize microbial-associated molecular patterns (MAMPs) and damage-associated molecular patterns (DAMPs). In humans, ten functional TLRs have been identified (TLR1–TLR10), whereas in mice, twelve (TLR1–TLR9 and TLR11–TLR13) [27]. Based on their predominant subcellular localization in immune cells, TLRs can be broadly categorized into two groups. TLRs located on the cell surface include TLR1, TLR2, TLR4, TLR5, TLR6, and TLR10, while TLR3, TLR7, TLR8, and TLR9 are primarily expressed within intracellular compartments such as endosomes, lysosomes, and the endoplasmic reticulum. Additionally, TLR2 can form heterodimers with TLR1, TLR6, and TLR10 on the cell surface. The endosomal TLRs primarily recognize nucleic acids derived from viruses and bacteria, while the cell surface TLRs detect various bacterial components such as lipopolysaccharides (LPS) [7], peptidoglycans (PGN) [28], flagellin [29], and lipoproteins [30]. Endogenous activators of TLRs include self-mRNA released from necrotic cells, mitochondrial DNA (mtDNA), high-mobility group box 1 (HMGB1), and heat shock proteins (Table 1).

**Figure 1:**
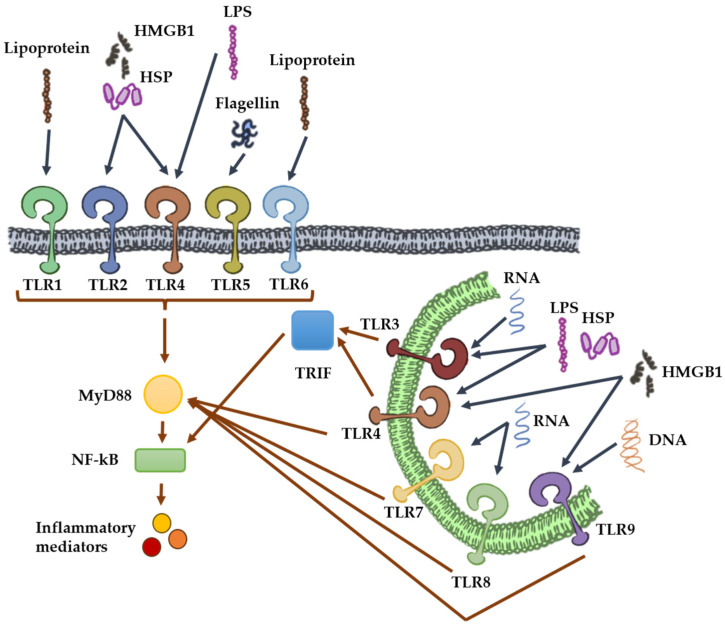
Diagram of Toll-like receptor (TLR) signaling pathways. TLRs located on the cell surface include TLR1, TLR2, TLR4, TLR5, and TLR6, while TLR3, TLR7, TLR8, and TLR9 are primarily expressed within intracellular compartments. TLRs detect MAMPs (viral and bacterial components like LPS and flagellin or nucleic acids) and DAMPs like self-mRNA, mtDNA, HMGB1, and heat shock proteins.

In immune cells, the activation of TLRs triggers a canonical signaling cascade that begins with the recruitment of the adapter protein myeloid differentiation primary response 88 (MyD88). This process leads to the activation of (nuclear factor kappa-light-chain-enhancer of activated B cells (NF-κB), which in turn promotes the transcription of cytokines and inflammatory genes [31,32]. However, TLR3 is an exception; it activates the adapter protein TIR-domain-containing adapter-inducing interferon-β (TRIF). In the case of TLR4, when located in endosomes, it can activate both MyD88 and TRIF. This dual activation results in the activation of the transcription factor IRF-3, which primarily enhances the transcription of type I interferons (Figure 1).

Additionally, TLR signaling can influence the activation of inflammasomes, particularly NLR family pyrin domain containing 3 (NLRP3), either directly or indirectly through NF-κB pathways. Inflammasomes are intracellular complexes that activate caspase-1, which processes pro-inflammatory cytokines such as interleukin-1 beta (IL-1β) and interleukin-18 (IL-18). Upon recognition of pathogen- or damage-associated molecular patterns (PAMPs or DAMPs), TLRs initiate signaling pathways that prime the expression of inflammasome components. This priming step is followed by the full assembly and activation of the inflammasome complex, culminating in the activation of caspase-1. The interplay between TLRs and inflammasomes serves to amplify and fine-tune immune responses to infection and cellular stress. While the roles of inflammasomes in peripheral immunity are increasingly well characterized, their functions within the CNS are only beginning to be elucidated, highlighting the need for further investigation [33,34].

TLRs are primarily expressed in immune cells; however, their expression extends to various cell types within the nervous system. In the peripheral nervous system (PNS), TLRs are found in sensory neurons, where they contribute to pain modulation, as well as in satellite glial cells and Schwann cells, where they play roles in neuroinflammatory responses following injury or infection. In the CNS, TLRs are predominantly expressed in microglia—the brain’s resident immune cells—but are also found in astrocytes, oligodendrocytes, and neurons. Although *TLR* expression in neurons is increasingly recognized, the precise signaling mechanisms and functional consequences in these cells remain incompletely understood. (for review, see [35]). Interestingly, they have been detected in the brain, with varying levels of protein and transcript expression at different ages, suggesting distinct roles during development and aging [36,37,38,39,40]. These findings suggest that TLRs may have functions beyond their immune roles, particularly in regulating various aspects of brain physiology, including emotional and cognitive domains, in both healthy and diseased states. Supporting this, several recent studies have highlighted the involvement of TLRs in diverse brain functions, including neurogenesis, neurodevelopment, synaptic plasticity, mood regulation, and memory. Moreover, TLRs have been implicated in diverse brain disorders, including anxiety, depression, autism spectrum disorder, attention deficit hyperactivity disorder (ADHD), Parkinson’s disease, and dementia.

## 3. Role of Toll-like Receptors in Emotional Regulation

Over the past decade, some studies have identified a role for Toll-like receptors (TLRs) in regulating emotions at both physiological and pathological levels, highlighting their relevance in a range of psychiatric disorders that involve dysfunction in emotional processes such as stress response, anxiety regulation, and mood stability (Table 2). Although TLR1 has been less studied in emotional regulation compared to other TLRs, one study found that its expression in peripheral blood mononuclear cells (PBMCs) from patients with depression was downregulated below baseline following antidepressant treatment [41]. Regarding TLR2, network analyses have identified it as a key hub gene implicated in both depression and suicidal behavior [42,43,44]. Interestingly, in non-medicated patients with major depressive disorder (MDD), *TLR2* mRNA expression was found to be significantly elevated in monocytes compared to healthy controls. This increase was normalized following antidepressant treatment, suggesting a potential link between TLR2 and immune dysregulation in depression [45]. Similarly, higher *TLR2* expression was observed in patients diagnosed with bipolar disorder and anxiety [46] and on Th17/Tc17-like cells in MDD patients with comorbid multiple sclerosis [47].

Compelling evidence indicates that early-life stressors play a significant role in the development of psychiatric and mood disorders later in life. Interestingly, a study found that carriers of the *TLR2* rs3804099 TT genotype may be more vulnerable to early stress and inflammation-mediated damage, potentially accelerating the earlier onset of bipolar disorder [145]. Moreover, rats subjected to maternal deprivation (MD) stress exhibited increased TLR2 expression along with altered cytokine and inflammatory mediator profiles. Specifically, the levels of IL-5, IL-6, IL-7, IL-10, tumor necrosis factor (TNF-α), and interferon gamma (IFN-γ) were elevated in the prefrontal cortex (PFC) of MD-exposed rats. Notably, TNF-α and IFN-γ remained elevated in infant rats, suggesting a persistent neuroimmune dysregulation linked to TLR2 during early-life stress [68].

Furthermore, an elegant study by Nie et al. identified TLR2/4 as a key mediator of repeated social defeat stress (R-SDS)-induced social avoidance—a well-established model of stress-induced depression—via microglial activation in the medial prefrontal cortex (mPFC). Notably, the loss of TLR2/4 abolished R-SDS-induced social avoidance and anxiety in mice while mitigating stress-induced neuronal response attenuation, dendritic atrophy, and microglial activation in the mPFC. Moreover, microglia-specific *TLR2/4* knockdown in the mPFC effectively blocked social avoidance, highlighting the critical role of TLR2/4 signaling in stress-related behavioral responses [66]. Similarly, increased HMGB1 levels and TLR2/4-dependent microglial activation were observed in the medial prefrontal cortex, while blocking HMGB1-RAGE signaling effectively attenuated social avoidance behaviors in a rodent model of chronic social defeat stress [67].

On the other hand, recent findings show that TLR2 deficiency increases susceptibility to learned helplessness, while *TLR2* activation with the agonist Pam3CSK4 reverses stress-induced impairments in sociability and novel object recognition tests, suggesting a protective role of TLR2 stimulation against depression-like states [69]. Similarly, in a rat model of chronic unpredictable stress, *TLR2* gene expression levels decreased in parallel with improvements in anxiety-like behavior and cognitive dysfunction in treated rats [61]. However, the absence of *TLR2* in mice is associated with increased anxiety levels [64].

Several studies have shown that *TLR3* expression levels are significantly elevated in the prefrontal cortex of postmortem brains of depressed suicide victims [43,80] and in the blood of depressed patients [41,81] compared to controls. Notably, these levels normalize following antidepressants [41], suggesting TLR3 as a potential biomarker of depression severity and treatment response. Similarly, TLR3 has been implicated with anxiety regulation. Mice lacking *TLR3* exhibit impaired amygdala-related behavior and anxiety-like behavior in the cued fear-conditioning, open field, and elevated plus maze tasks [78]. However, in young mice, *TLR3* deletion appears to have anxiolytic-like effects [79]. In line with this, other findings showed that activation of *TLR3* by poly(I:C) administration induces depressive and anxiety-like behaviors in rats [92]. Furthermore, a potential interaction between TLR3 signaling and the endocannabinoid system in anxiety regulation has been suggested. TLR3 mediated microglial activation and anxiety-like behaviors in female mice that were suppressed by the fatty-acid amide hydrolase 1 (FAAH) inhibitor URB597 [83]. Moreover, chronic ethanol exposure upregulated *TLR3* and *NF-κB* expression in the hippocampus, leading to neuroinflammation and anxiety-like behavior. Notably, silencing *TLR3* attenuated these effects, suggesting a pivotal role for TLR3 signaling in ethanol-induced neuroinflammation and its contribution to anxiety modulation [82].

TLR4 is one of the most extensively studied members of the TLR family in psychiatry, and numerous studies have highlighted its significant role in emotional regulation. Genetic studies in humans evidenced that *TLR4* single nucleotide polymorphisms are associated with anxiety, suicidal behavior, and other symptoms in patients with first-episode depression [97]. In experimental models, while TLR4 does not regulate baseline GABAergic transmission in the central amygdala, stress-induced enhancement of GABA release by corticotropin-releasing factor (CRF) is absent in *TLR4* knockout rats [106]. Further studies have shown that *TLR4* knockout (KO) mice exhibit increased anxiety-like behavior and reduced social interaction compared to wild-type control mice [104]. Imaging studies using Positron Emission Tomography in chronic social defeat stress (SDS) models have revealed changes in both *COX-1* and *TLR4* expression [95]. Repeated SDS activates microglia in the medial prefrontal cortex via TLR2/4, inducing neuronal atrophy and social avoidance through the release of IL-1α and TNF-α [66]. Moreover, increased TLR4 protein levels in the hippocampus were associated with behavioral despair, social avoidance, and anxiety-like behaviors, which were normalized with fluoxetine treatment, direct TLR4 blockade, or genetic deletion [99].

Stress activates TLR4 signaling in the prefrontal cortex (PFC), triggering NF-κB activation and the expression of pro-inflammatory enzymes such as nitric oxide synthase and cyclooxygenase-2. This cascade leads to oxidative and nitrosative damage, which is attenuated in models with defective TLR4 signaling [96]. Notably, pre-treatment with the TLR4 inhibitor TAK-242 prior to stress exposure reduces neuroinflammation, highlighting the potential of TLR4 blockade as a therapeutic strategy for stress-related neuropsychiatric disorders [98].

Interestingly, TLR4 signaling also has sex-specific effects. Deletion of *TLR4* in Tph2-expressing serotonergic neurons reduces anxiety-like behavior in male mice but not in female mice. Targeted inhibition in the dorsal raphe nucleus reverses stress-induced anxiety following chronic immobilization, likely by altering serotonin synthesis, reuptake, and transmission [103]. Microglial *TLR4* ablation significantly reduces the persistent depression-like behavior characteristic of female mice [102]. Moreover, studies on predator odor exposure studies in *TLR4* knockout rats show that female rats exhibit altered affective responses compared to their wild-type counterparts, highlighting the involvement of TLR4 in stress-induced affective regulation in a sex-specific manner [105].

The non-immune roles of TLR5 in the context of psychiatry have been studied only to a limited extent. One of the earliest indications of TLR5 involvement in depression came from a clinical study reporting increased *TLR5* expression in the peripheral blood of patients with major depressive disorder [81], which was normalized after antidepressant treatment [41]. More recently, a rodent study found that mice lacking *TLR5* displayed reduced basal anxiety, along with an altered hypothalamic–pituitary–adrenal (HPA) axis response to acute restraint stress. These mice also showed decreased plasma corticosterone levels and reduced c-fos expression in the hypothalamic paraventricular nucleus, suggesting a role for TLR5 in modulating neuroendocrine responses to stress [118]. Notably, depression- and cognition-related behaviors did not differ between TLR5 knockout (KO) and wild-type (WT) mice. Additionally, there were no significant changes in the expression of key cytokines (*IL-6*, *IL-10*, *TNF-α*) or other TLRs (*TLR2*, *TLR3*, *TLR4*) in the prefrontal cortex, amygdala, or hippocampus of *TLR5* KO mice compared to WT controls [118].

Regarding TLR6, only one study found that although *TLR6* mRNA levels in peripheral blood mononuclear cells (PBMCs) were unchanged in patients with depression [81], they dropped below levels seen in healthy controls following antidepressant treatment [41]. The same studies reported increased *TLR7* expression in patients with MDD, which returned to normal levels after antidepressant treatment, indicating a potential role for TLR7 in emotional regulation. Findings from several rodent studies further support this involvement. Mice lacking *TLR7* spend more time in the open arms of the elevated plus maze and the light compartment of the light–dark box, indicating a reduction in anxiety-like behavior. They also exhibit lower levels of territorial and social aggression, suggesting a complex role for *TLR7* in balancing neurodevelopmental and behavioral outcomes [127]. Social isolation in rats increased the gene expression levels of *TLR7*, *MyD88*, and *TRAF6* mRNA in the hippocampus compared to socially housed controls. Acute treatment with the antidepressant fluoxetine and the anti-inflammatory drug etanercept effectively reversed these molecular changes [122]. TLR7 activation also has been shown to induce sickness behavior with sex-dependent metabolic and behavioral effects in the prefrontal cortex [146].

Interestingly, maternal immune activation induced by the TLR7 agonist resiquimod during mid-gestation increased offspring susceptibility to blood–brain barrier leakage after puberty and led to elevated plasma corticosterone levels 60 min after restraint stress in both males and females, suggesting an impaired hypothalamic–pituitary–adrenal (HPA) axis stress reactivity in adult offspring [128]. Furthermore, TLR7/8-driven maternal immune activation (MIA) elevated placental and fetal brain cytokines in juvenile and adult mice, contributing to delays in developmental milestones. Both juvenile and adult male and female MIA offspring exhibited reduced social-like behavior in social interaction tests. Notably, anhedonia-like behavior was more pronounced in adult female MIA mice, as indicated by the sucrose preference test, where resiquimod-exposed females showed significantly lower normalized sucrose intake compared to vehicle-treated controls [129]. In addition, inhibition of TLR7-mediated inflammatory signaling, previously activated by imiquimod, has shown efficacy in models of postpartum depression [130].

Although limited, a few studies have also begun to associate TLR8 with emotional regulation. DNA methylation is increasingly recognized as a key epigenetic mechanism through which chronic stress may exert long-lasting effects on gene expression and function. In the context of post-traumatic stress disorder (PTSD), a recent study reported altered gene-specific DNA methylation patterns, particularly involving the *TLR8* gene in patients with PTSD related to childhood abuse [147]. However, a separate study conducted on military personnel with similarly reported childhood trauma did not observe methylation changes in *TLR8* [148], suggesting that specific environmental factors, population characteristics, or ethnic background may influence these epigenetic effects. Collectively, these findings support a potential role for TLR8 in mediating the enduring biological effects of psychosocial stress on emotional outcomes, linking innate immune signaling with stress-related epigenetic regulation.

Failure of the innate immune system to properly engage TLR9 is associated with anxiety-related inflammation, and peripheral administration of specific TLR9 oligonucleotide activators has been shown to prevent post-traumatic inflammation and anxiety in stressed mice [137]. Moreover, *TLR9* deficiency confers resistance to chronic stress-induced lymphocyte apoptosis and blocks the imbalance of Th1/Th2 cytokine levels, suggesting that TLR9 is a key mediator of chronic stress-induced immune suppression [140]. In models of chronic unpredictable mild stress (CUMS) combined with corticosterone exposure, upregulation of *TLR9* in the medial prefrontal cortex is associated with depressive and anxiety-like behaviors, whereas inhibition of *TLR9* signaling by blocking cell-free mitochondrial DNA (cf-mtDNA) markedly attenuates stress-induced social behavioral deficits [138,139].

Overall, these studies highlight Toll-like receptors as important regulators of emotional processes relevant to psychiatric disorders. Their influence on mood, anxiety, and stress responses underscores a critical link between innate immune signaling and emotional dysregulation. However, further research is needed to elucidate the specific mechanisms underlying their effects on emotional responses.

## 4. Implications of TLRs in Cognition and Memory

Numerous emerging studies highlight the role of certain TLRs in regulating cognitive and memory functions, influencing both normal brain processes and the pathophysiology of psychiatric disorders characterized by deficits in learning, memory, and executive functions (Table 2). *TLR2* KO mice show memory spatial deficits in the Morris water maze, reduced locomotor activity, and inhibited long-term potentiation (LTP) [64]. Similarly, Park et al. reported that *TLR2* KO mice exhibit spatial memory deficits in the Barnes maze test [65]. Notably, while *TLR2* KO mice could eventually learn the Barnes maze paradigm through repetitive training, their memory retrieval and reversal learning abilities improved at a slower rate compared to WT animals [63], suggesting that *TLR2* KO mice may be a model for studying schizophrenia-associated cognitive impairments.

Furthermore, transcriptomic analyses of microglia in the dentate gyrus of the mouse hippocampus revealed an overexpression of the autophagy-related gene *ATG7* in fear engram cells, which in turn upregulated *TLR2/4* mRNA expression in dentate gyrus (DG) microglia. Knocking down microglial *TLR2/4* rescued fear memory destabilization induced by *ATG7* overexpression or *Rac1* activation in DG engram cells, highlighting a Rac1-driven engram–microglia crosstalk mediated by ATG7 and TLR2/4 in the destabilization of contextual fear memory [59]. Postnatal TLR2 activation with specific ligands (Pam3CSK4 or FSL-1) produces heterodimer-specific effects on adult cognition. TLR2/6 activation enhances motor and fear learning, whereas TLR2/1 activation impairs spatial learning and amplifies fear memory. Conversely, developmental *TLR2* deficiency results in spatial learning deficits and heightened fear responses [60]. These findings suggest that adult cognitive behavior may be shaped, at least in part, by early-life activation or alterations in the TLR2 pathway.

Postoperative cognitive dysfunction (POCD) is characterized by a decline in cognitive function, particularly in memory and executive functions, that can persist for months to years after surgery. In some cases, POCD may last several years following major surgical procedures and is considered a significant risk factor for dementia later in life [149,150]. Evidence suggests that stress and systemic inflammation triggered by surgery and/or anesthesia play a key role in memory deterioration [151]. Rodent studies have demonstrated alterations in hippocampal inflammation and synaptic plasticity, leading to cognitive deficits, as well as impaired neuronal–astrocyte metabolic coupling in the hippocampus [152]. Remarkably, in a POCD model, surgery induced neuroinflammation and cognitive impairment in C57BL/6J mice but not in *TLR2*^−^/^−^ mice or those treated with a TLR1/TLR2 antagonist.

Additionally, surgery resulted in increased TLR2 protein levels in the hippocampus and elevated HMGB1 levels in the nuclei of the cerebral cortex and hippocampal cells. These effects were reversed by an HMGB1 antagonist [62], suggesting that HMGB1 upregulates *TLR2* expression in the hippocampus after surgery, contributing to cognitive decline. Another study found that isoflurane inhalation in neonatal rats resulted in increased cell apoptosis, inflammation, activation of the TLR2/NF-κB signaling pathway, decreased PSD95 expression, and impaired spatial learning and memory abilities in the Morris water maze. Dexmedetomidine, a selective α2-adrenergic receptor agonist with sedative, anxiolytic, analgesic, and anesthetic properties, improved spatial learning and memory impairment associated with isoflurane-induced neurotoxicity by inactivating the TLR2/NF-κB pathway—an effect that was reversed by the TLR2 agonist Pam3CSK4 [58].

Deletion of TLR3 in young mice has been shown to reduce spatial learning deficits. However, working memory appears unaffected, whereas *TLR3*-deficient adult mice exhibit enhanced hippocampal-dependent working memory, increased CA1 and dentate gyrus volumes, increased neurogenesis, and increased activation of *ERK* and *CREB*-suggesting that constitutive TLR3 signaling usually suppresses hippocampal plasticity [78,79].

In models combining interferon-α with poly(I:C), co-delivery synergistically induces pro-inflammatory gene expression in the hippocampus and prefrontal cortex, reduces apical dendritic spine density, downregulates TrkB signaling, decreases levels of VGLUT-1 and PSD95, and increases AMPAR1 expression, which collectively impairs neuronal excitability and suggests a mechanism for IFN-associated depression [74,75].

In cultured hippocampal and prefrontal neurons, co-stimulation with murine IFN and poly(I:C) activates *Stat1* and *Stat3*, induces the expression of pro-inflammatory cytokines, selectively upregulates downstream interferon regulatory and NF-κB-related genes, and promotes neuronal apoptosis, further linking TLR3 activation to impaired neuroplasticity [76]. In adolescent rats subjected to maternal separation, a single peripheral administration of poly(I:C) robustly activates TLR3, IL-6, and NF-κB in the medial prefrontal cortex, resulting in working memory impairments without inducing oxidative stress [72].

In models of chronic neuropathic pain, increased *TLR3* expression in hippocampal neurons, accompanied by increased extracellular double-stranded RNAs, correlates with impaired memory, increased inflammatory cytokine release, and enhanced neuronal apoptosis. Conversely, *TLR3* knockdown or inhibition improves cognitive outcomes [77].

Early postnatal exposure to poly(I:C) induces persistent TNF-α-mediated neuroinflammation and alters the expression of memory-related genes (e.g., BDNF, Arc, EGR1) in the frontal cortex and hippocampus, resulting in long-term impairments in spatial and fear conditioning memory [73]. In neonatal inflammatory responses following febrile seizures, characterized by increased expression of *TNF-α*, *IL-1β*, and *TLR4*, resulting in adult memory deficits that can be alleviated by pretreatment with TAK-242, which reduces inflammatory cytokines and improves cognitive performance [91]. Surgical models, such as splenectomy in aged mice, demonstrate that increased hippocampal TLR4 signaling is correlated with spatial cognitive deficits and neuroinflammation. Fluoxetine pretreatment partially restores cognitive function by downregulating *TLR4*, *MyD88*, and *phosphorylated NF-κB p65* in microglia, whereas preoperative intracerebroventricular LPS injection attenuates the efficacy of fluoxetine [153]. Furthermore, intracerebral infusion of a TLR4 antagonist in adult mice alters anxiety responses and impairs the developmental regulation of spatial reference memory and fear learning, but does not significantly alter hippocampus-dependent cognitive behavior [93].

In addition, recent studies have shown that binge-like ethanol exposure during adolescence in rats induces neuroinflammation through TLR4 signaling, leading to impairments in synaptic plasticity and cognitive function. Forty-eight hours after two binge episodes, rats showed impairments in novel object recognition, reduced long-term depression (LTD), enhanced long-term potentiation (LTP), and increased sensitivity of excitatory neurotransmission to an antagonist of the GluN2B subunit of the NMDA receptor. Notably, these deficits were prevented by co-administration of ethanol with a TLR4 antagonist, suggesting that TLR4-mediated neuroimmune responses play a key role in these alterations [90]. Immunolabeling analyses further revealed a reduction in neuronal *TLR4* expression at 48 h, a change that was counteracted by minocycline pretreatment, while microglial reactivity and mRNA levels of inflammatory cytokines remained unchanged. Further supporting this administration of lipopolysaccharide was the loss of doublecortin, accompanied by increased expression of cleaved caspase 3 and pNF-κB p65 in the hippocampal dentate gyrus, which mimicked ethanol-induced loss of neurogenesis. Taken together, these findings suggest that interventions targeting TLR4 may protect against impairments in synaptic plasticity and memory deficits associated with neuroinflammation [94].

Interestingly, a recent study demonstrated that stimulating Toll-like receptor 5 (TLR5) through mucosal delivery of a flagellin-containing fusion protein contributes to extended health and lifespan in both male and female mice, as evidenced by several signs, including enhanced cognitive capacity [154].

Although very few studies have explored the role of TLR7 in memory and cognition, evidence suggests that TLR7 may contribute to the formation of contextual fear memory, possibly through the induction of type I interferon and IL-1β [124] as well as to exercise-induced contextual learning [126]. Furthermore, *TLR7* upregulation in the hippocampus, accompanied by elevated levels of let-7b, has been implicated in perioperative neurocognitive disorders, where it contributes to memory impairment [125]. However, another study reported that deletion of *TLR7* alters expression profiles of genes related to neural function, hippocampal long-term potentiation, and contextual memory [127]. In cultured cortical neurons, TLR8 activation inhibits neurite outgrowth and induces apoptosis, independently of the canonical TLR–NF-κB signaling pathway [155], suggesting a distinct, non-immune role for TLR8 in neuronal development. *TLR8* expression has also been identified in hippocampal interneurons in a subregion- and cell-type-specific manner. While calretinin-positive interneurons exhibit minimal *TLR8* expression, parvalbumin-positive interneurons consistently co-express *TLR8* across all hippocampal subregions. In contrast, somatostatin-positive interneurons co-expressing TLR8 are primarily localized in CA3, with limited expression in CA1 and the dentate gyrus [156]. These patterns suggest that TLR8 may contribute to the regulation of hippocampal functions, including memory, emotional processing, and seizure susceptibility, and highlight the need for further research into its role in neural circuit development and function.

Stimulation of TLR9 via CpG ODNs has been shown to improve cognitive performance and reduce cerebral amyloid angiopathy (CAA) without associated toxicity [134]. In models of Alzheimer’s disease, long-term use of the class B CpG ODN 2006 induces a favorable degree of innate immune stimulation that ameliorates both CAA and tau pathology while improving behavioral outcomes in aged subjects [157]. In parallel, a subset of excitatory hippocampal CA1 neurons undergoing persistent double-stranded DNA damage activates TLR9 signaling, which is critical for centrosome function, DNA damage repair, and perineuronal network assembly. Neuron-specific knockdown of *TLR9* impairs memory formation, highlighting its crucial role in recruiting damaged neurons into memory circuits [136]. In addition, inhibition of *TLR9* in hippocampal neurons blocks NMDA-induced activation of caspase-3 and reduction of cell surface AMPA receptors, suggesting that TLR9 signaling contributes to long-term chemical depression of synapses [158]. Spatial memory deficits observed in CpG-treated wild-type mice but not in TLR9-deficient counterparts further underscore that exposure to bacterial DNA via TLR9 can impair neuronal function, promote neuroinflammation, and lead to neurodegeneration [135].

TLR9 has also been implicated in sensorimotor and autonomic functions. *TLR9*-deficient mice exhibit a hyper-responsive sensory and motor phenotype, including hypersensitivity to thermal stimuli, increased motor responsiveness under anxiety-inducing conditions, and synaptic abnormalities, which together suggest that TLR9 is important for the proper development of sensory, motor, and neuromuscular junction (NMJ) function [159,160]. Deletion of *TLR9*, along with *TLR3*, has been associated with impaired motor performance and alterations in working memory, suggesting that TLR9 contributes to a wide range of central nervous system functions beyond its immunological role [79].

## 5. Metabolic Regulation

Growing evidence supports a complex and bidirectional crosstalk between the immune system, metabolism, and the brain, with critical implications for psychiatric and metabolic disorders. Chronic low-grade inflammation—a hallmark of conditions such as obesity, type 2 diabetes, and metabolic syndrome—is increasingly recognized as a contributing factor to mood disorders, including depression and anxiety [161,162]. Although this relationship remains underexplored, several studies have highlighted the impact of TLRs on metabolic homeostasis and neural function (Table 2).

Recent studies suggest that adipocytes may play a crucial role in the physiological regulation of immune responses within fat depots through Toll-like receptor (TLR) signaling pathways. Obesity induced by a high-fat diet or leptin deficiency has been shown to upregulate the expression of TLR1–9 and TLR11–13 in murine adipose tissues [143]. Notably, the extent of upregulation of TLR1, TLR4, TLR5, TLR8, TLR9, and TLR12, along with most downstream signaling molecules and target cytokine genes in visceral adipose tissue, was greater in diet-induced obese (DIO) mice compared to ob/ob mice. These findings suggest that TLRs overexpressed in expanded adipose tissue may contribute significantly to the development of obesity-associated meta-inflammation. In line with this, increased TLR2 expression levels have also been reported in the peripheral blood of both obesity and diabetes subjects [163,164,165].

Furthermore, treatment with alpha-lipoic acid and metformin has been shown to improve insulin resistance and cognitive deficits by modulating TLR2 signaling, further supporting the involvement of TLR2-driven inflammation in metabolic dysfunctions and their neurological consequences [48]. Additionally, mice lacking TLR2 are protected from diet-induced adiposity, insulin resistance, hypercholesterolemia, and hepatic steatosis, suggesting that TLR2 plays a critical role in the development of diet-induced metabolic syndrome [53]. Other studies have similarly reported that TLR2-deficient mice show improved insulin sensitivity and enhanced hepatic insulin signaling [54] and are protected from high-fat diet-induced insulin resistance and pancreatic beta cell dysfunction [55]. Conversely, another study identifies TLR2 as playing a role in the hypothalamic regulation of metabolism and protection against obesity. TLR2-deficient mice develop age-related obesity and show increased susceptibility to HFD-induced weight gain [51]. The study further reported that TLR2 levels increase with age or exposure to a high-fat diet (HFD), specifically in pro-opiomelanocortin (POMC) neurons of the arcuate nucleus, a key hypothalamic region involved in central metabolic regulation. Interestingly, obesity persisted in chimeric mice possessing TLR2-positive immune cells, suggesting that the observed effects are mediated through non-immune mechanisms [166].

TLR3 may also play a role in regulating metabolic homeostasis. In hyperplastic adipose tissue, decreased TLR3 expression has been linked to metabolic inflammation, suggesting a potential role in systemic inflammatory imbalance [71]. Under inflammatory conditions, astrocytes—which are particularly sensitive to TLR3 ligands such as poly(I:C) and HMGB1—upregulate TLR3 expression, produce IFN-β, and modulate their energy metabolism through autophagy to regenerate damaged mitochondria. These mechanisms, in turn, may influence the excitability of hippocampal neurons [70].

A growing body of evidence highlights the critical role of TLR4 signaling in linking metabolic, inflammatory, and neuropsychiatric processes. Mice lacking TLR2 or TLR4 have reduced basal heart rate, altered thermoregulatory responses, and changes in food intake and body mass [56]. Moreover, high-fat diet fed mice display decreased TLR2 and increased TLR4 mRNA expression in the hippocampal tissue and anxiety- and depressive-like behaviors compared to lean-fed mice [51]. Chronic exposure to a diet characterized by very low n-3 PUFA content results in higher expression of TLR2 and TLR4 in the hippocampus of female-treated rats and is associated with depressive-like symptoms and higher susceptibility to stress [49]. Supplementation with n-3 polyunsaturated fatty acid reduces depressive-like behaviors by lowering TLR4 expression and attenuating hippocampal neuroinflammation in male mice [100]. Moreover, adipose-derived mesenchymal stem cells protect against chronic mild stress-induced depressive behaviors by activating the Nrf2/HO-1 pathway and suppressing the TLR4/NF-κB signaling pathway [101]. Experimental models combining lipopolysaccharide (LPS) administration with chronic mild stress have demonstrated that TLR4 modulates mitochondrial biogenesis in the hippocampus through the PGC1-α/NRF1/Tfam axis. This activation is accompanied by increased expression of MyD88, NF-κB, and TNF-α, along with elevated hippocampal energy metabolism and phosphorylation of AMP-activated protein kinase (AMPK). Notably, treatment with fluoxetine, pentoxifylline, or their combination effectively attenuates these alterations, suggesting that inhibition of the TLR4/NF-κB pathway may enhance mitochondrial biogenesis and neuronal function independently of AMPK activation [84]. Supporting this, pre-diabetic mice exhibit elevated TLR4 expression and increased activity of the alternative NF-κB pathway [85]. Similarly, the insulin receptor sensitizer dicholine succinate has been shown to prevent TLR4 upregulation and related affective disturbances induced by a high-cholesterol diet [89].

In models of type 2 diabetes, exercise training reduces inflammation in the dentate gyrus through the irisin/TLR4/MyD88/NF-κB pathway, enhancing hippocampal neurogenesis and memory performance [167]. Furthermore, diabetic and non-diabetic rats exposed to chronic stress develop depressive-like behavior, with more pronounced symptoms in diabetic animals. These behavioral changes are associated with vascular and metabolic dysfunctions, which include an increase in aortic expression of TLR4 and pro-inflammatory cytokines, such as TNF-α and IL-1β. Chronic treatment with antidepressants like fluoxetine and imipramine reverses these metabolic and inflammatory abnormalities, ameliorating both affective and physiological disturbances [86]. Consistently, another study shows that diabetic mice exhibit metabolic disturbances alongside cognitive deficits and heightened anxiety-like behavior. These impairments were markedly reduced in TLR4 knockout mice, which also showed changes in the expression of enzymes related to brain energy metabolism [87].

In humans, a nonsense polymorphism (R392X) in the TLR5 gene has been associated with protection against obesity while predisposing carriers to diabetes [110]. In rodents, the constitutive absence of TLR5 results in notable disruptions to lipid metabolism and the circadian rhythm, further indicating a critical role for TLR5 in maintaining metabolic and physiological homeostasis [111]. Older TLR5-deficient non-obese diabetic (NOD) mice have an increased risk of developing spontaneous type 1 diabetes compared to wild-type controls [112]. Furthermore, when challenged with a low-fat diet, wild-type C57BL/6 mice display a marked reduction in adipocyte size within epididymal fat. This response is absent in TLR5-deficient mice, as well as in TLR2^−^/^−^ and TLR4^−^/^−^ models [57]. In addition, TLR5 gene knockout has been shown to impair some of the beneficial effects of weight loss in diet-induced obesity models [113].

In models of gestational diabetes mellitus, increased immune markers, including TLR4, TLR5, IL-22, and IL-23, have been detected in the placenta, suggesting a role for TLR4 and TLR5 in the inflammatory processes associated with maternal metabolic disturbances [88]. TLR6 is significantly overexpressed in hepatocytes from NAFLD morbidly obese patients compared to non-obese patients, suggesting that deregulated TLR6 may potentiate liver inflammation in obesity [52,119]. Toll-like receptor 7 (TLR7) is expressed in adipocytes and regulates adipocyte function [168]. TLR7 activation has been shown to exacerbate high-fat diet-induced hyperinsulinemia, dysglycemia, and lupus autoimmunity [120,121,123]. Moreover, TLR7 stimulation in CD8^+^ T cells enhances glycolysis via the AKT-mTOR-IRF4 axis [169]. Interestingly, strenuous exercise has been reported to reduce TLR7-mediated production of TNF-α and IFN-α [170].

A recent study identified increased expression of IL-2 in adipose tissue as a potential novel biomarker for the progression of metabolic inflammation and insulin resistance in obese individuals. Notably, IL-2 upregulation correlated with increased expression of TLR2, TLR8, and TLR10, suggesting that these receptors may contribute to the establishment and maintenance of an inflammatory environment that promotes IL-2 expression and metabolic dysfunction [171]. In line with this, TLR8 signaling has been shown to mediate metabolic reprogramming, particularly in immune cells. In various experimental models, including cancer immunotherapy settings, TLR8 activation has been linked to altered glucose metabolism in human regulatory T cells (Tregs) and CD4^+^ T cells [172,173,174]. Additionally, in vascular smooth muscle cells, miR-378a has been shown to inhibit fatty acid-induced proliferation, migration, and inflammation by targeting both IGF1 and TLR8, further implicating TLR8 in lipid-related metabolic processes [175].

TLR9 activation may play a dual role in metabolic homeostasis. In diabetic conditions characterized by chronic hyperglycemia, high glucose induces TLR9 activation in astrocytes, leading to reactive oxygen species (ROS) generation that impairs thrombospondin-1 (TSP-1) secretion and contributes to synaptic protein loss; both TLR9 deficiency and antioxidant treatment restore TSP-1 levels, implicating TLR9-mediated ROS signaling in metabolic inflammation and synaptic degeneration [133]. Additionally, TLR9 modulates cellular energy metabolism in both cardiomyocytes and neurons by reducing energy substrates and increasing the AMP/ATP ratio, thereby activating the AMP-activated protein kinase (AMPK) [131]. In skeletal muscle, TLR9 interacts with the autophagy protein Beclin 1 to coordinate exercise-induced AMPK activation, GLUT4 translocation, and glucose uptake, as mice lacking TLR9 are deficient in these metabolic responses [132].

Additional studies have highlighted the involvement of TLR9 in neuroprotective autophagy. In hypoxic-ischemic encephalopathy (HIE), treatment with CpG-ODN increases the phosphorylation of AMPK and its downstream targets (including ULK1, AMBRA1, LC3II/I, and LAMP1) and inhibition of TLR9 or AMPK reverses these effects, leading to decreased autophagy and poorer neurobehavioral outcomes [132]. Conversely, TLR9 activation can be protective by modulating autophagic processes; however, its prolonged activation under stressful conditions, such as exposure to bacterial CpG DNA, may impair spatial memory and promote neurodegeneration [135].

Although there is no conclusive data on the role of TLR10, TLR11, or TLR12 in emotional or cognitive processes, some studies have suggested their involvement in metabolic regulation. In particular, obese individuals carrying TLR10 polymorphisms exhibit reduced macrophage infiltration in adipose tissue, lower leptin levels, and higher adiponectin levels, whereas no such differences are observed in healthy subjects [142]. Moreover, TLR10 gene and protein expression are markedly upregulated in obesity and type 2 diabetes, correlating with body mass index. Additionally, reactive oxygen species (ROS) stimulate TLR10 expression in monocytic cells and peripheral blood mononuclear cells (PBMCs) through NF-κB/MAPK signaling and endoplasmic reticulum stress. Treatment with H_2_O_2_ and palmitate synergistically enhances TLR10 and pro-inflammatory cytokine expression, suggesting its potential as an immune marker and therapeutic target for metabolic inflammation [141]. Dietary interventions, such as quinoa supplementation, downregulate TLR11 and TLR12 expression while upregulating genes involved in lipid metabolism, thereby mitigating hepatic lipid accumulation and oxidative stress [144].

## 6. Gut Microbiome

In recent years, the intimate relationship between mental and metabolic health, as well as our intestinal flora, has been demonstrated; however, the molecular pathways underlying this communication are not yet fully understood. Here, we investigate the critical role of Toll-like receptors in gut–brain communication and how alterations in the microbiota can affect the expression of these receptors, leading to emotional, cognitive, and metabolic changes.

Dysbiosis, an imbalance in the gut microbiota, has been linked to mental health disorders, including anxiety and depression. Environmental toxins, such as arsenic, can induce significant dysbiosis by enriching Gram-negative bacteria and compromising intestinal barrier integrity, leading to an increased level of lipopolysaccharide (LPS) in the bloodstream, which triggers systemic inflammation, alters neurotransmitter levels, activates microglia, and disrupts brain architecture—factors that collectively contribute to anxiety- and depression-like behaviors in a dose-dependent manner [176]. Fecal microbiota transplantation (FMT) from arsenic-exposed mice to healthy recipients reproduces these neurotoxic effects, and suppression of *TLR4* using in vivo morpholino oligomers can restore normal parameters, demonstrating the direct involvement of TLR4 in the arsenic-gut–brain axis.

Similarly, substances like methamphetamine can also disrupt gut homeostasis, albeit through distinct mechanisms. Methamphetamine abuse disrupts gut homeostasis by activating TLR4-related colonic inflammation and reducing microbiota-derived short-chain fatty acids (SCFAs). Fecal transfers from methamphetamine-treated mice have been shown to mediate colonic inflammation and reproduce anxiety- and depression-like behaviors in recipients, highlighting the crucial role of gut microbiota in maintaining mental health [177].

Dietary components also influence the interplay between TLR signaling and the gut microbiota. Dietary fiber, for instance, modulates the TLR4/NF-κB signaling pathway by maintaining a balanced microbial community. Fiber intake reduces harmful bacteria, such as *Desulfovibrio*, while enriching beneficial populations like *Akkermansia* and *Ruminococcus*. This microbial shift not only attenuates oxidative stress and inflammatory responses via pathways including pyrimidine and tryptophan metabolism but also has a preventive effect on anxiety-like behavior in hyperuricemic mice [178].

Building on this, probiotic interventions help restore homeostasis by modulating TLR-mediated responses. For instance, treatment with *Bifidobacterium pseudocatenulatum* in obese mice reduces *TLR2* upregulation in the intestine and hippocampus. This treatment helps normalize serotonin levels and decreases depressive-like behavior. Additionally, butyrate supplementation enhances memory and cognitive functions by suppressing microglia-mediated neuroinflammation through the GPR109A/PPAR-γ/TLR4-NF-κB pathway [50,179]. Following probiotic intervention effects on mental health, recent studies reveal sex-specific effects of pubertal probiotic exposure on LPS-induced behaviors: it mainly reduces depression-like behavior in females, whereas, in males, it decreases anxiety-like behavior and stress reactivity [180]. Supporting these findings, LPS increases anxiety-like behavior in male wild-type mice (but not in Tlr4^−^/^−^ mice), and blocking the TLR4 TRIF pathway with (+)-naloxone produces opposite behavioral effects in males and females, with differences also observed in gut interleukin-6 expression [105,181]. Significantly, pubertal probiotic treatment prevented later-life LPS-induced impairments by modifying gut microbiota composition, reducing acute inflammation, and blocking stress-induced *TLR4* upregulation in the paraventricular nucleus (PVN) [180]. Overall, these findings highlight the long-term benefits of early probiotic interventions and the critical role of considering sex-specific differences in TLR4-mediated stress responses.

Irritable bowel syndrome (IBS)—a common and potentially disabling functional gastrointestinal disorder—has been linked to psychological issues, including anxiety and depression. Studies indicate that IBS patients face a three-fold increased risk of experiencing anxiety or depression compared to healthy individuals [182]. Patients with IBS exhibit alterations in TLR expression, including elevated TLR4 levels and changes in barrier-related genes, which are associated with impaired intestinal barrier function, specific gut microbial profiles, and disrupted tryptophan metabolism [183,184]. These findings suggest that modulation of TLR signaling could serve as a novel therapeutic target for managing both gastrointestinal and psychological symptoms in IBS.

Alterations in TLR5 sensing of the gut microbiome have been linked to the development of metabolic syndrome in *TLR5*^−^/^−^ mice [114] and disrupted gut microbiota composition, hyperphagia, hyperlipidemia, hypertension, insulin resistance, and increased adiposity [115]. At the same time, some other studies report that *TLR5*-deficient mice from different animal colonies do not exhibit overt basal inflammatory disease or metabolic abnormalities [116]. However, further research using different *TLR5*-deficient models suggests that these metabolic abnormalities are primarily driven by environmental changes affecting the microbiota rather than *TLR5* deficiency itself [117]. Supporting the involvement of TLR5 pathways, *Clostridium* cluster XIV bacteria have been implicated in the development of obesity via TLR5 signaling [108]. Similarly, recent findings show that flagellin—a component of gut bacteria such as *Enterobacteriaceae*—is enriched in individuals with type 2 diabetes (T2D), where it triggers a TLR5-mediated pro-inflammatory response in pancreatic islets, leading to beta-cell dysfunction characterized by impaired insulin production and secretion [107]. Exercise physiology has also revealed an intriguing role for TLR5. Exercise upregulates the production of TNF-α in response to flagellin through increased expression of *TLR5* on the intestinal cell surface, a process mediated by β-adrenergic receptor stimulation during exercise [109]. 

Collectively, these findings underscore that the gut microbiota and TLR signaling form a critical axis that regulates systemic inflammation, which in turn may influence emotional behavior, cognitive function, and metabolic homeostasis (summarized in Table 3). Understanding these intricate interactions is essential for developing targeted interventions that restore microbial balance and modulate TLR-driven inflammatory pathways, ultimately mitigating the detrimental effects associated with neuropsychiatric and metabolic disorders.

## 7. Conclusions

Toll-like receptors (TLRs) have emerged as key mediators of the dynamic crosstalk between the immune system, metabolic regulation, and central nervous system (CNS) function. Their widespread expression across peripheral immune cells and resident brain populations—including microglia, astrocytes, and neurons—enables TLRs to transduce systemic inflammatory and metabolic signals into region- and cell-specific changes in neurogenesis, synaptic plasticity, and neuronal survival. Beyond their role in immune defense, TLRs are essential for regulating normal physiological functions in the brain including emotional responses and cognition. Persistent or dysregulated TLR activation fosters a pro-inflammatory environment, disrupts mitochondrial function, and drives oxidative stress, mechanisms increasingly recognized as central to the pathogenesis of psychiatric disorders. 

Future studies should aim to elucidate the specific molecular mechanisms and cell-type-specific functions of TLR signaling to better understand the divergent outcomes of TLR activation across brain and peripheral tissues. Incorporating data on genetic polymorphisms and sex-specific differences will be essential for capturing individual variability in these responses. Ultimately, advancing our knowledge of TLR-driven immuno-metabolic crosstalk and its influence on neuroplasticity will be key to refining therapeutic strategies, potentially opening new avenues to alleviate the burden of psychiatric disorders.

## Figures and Tables

**Table 1:** Summary of known Toll-like receptor (TLR) ligands, chemical nature, and biological origin.

TLR	Ligand	Chemical Nature	Origin	Pathway
TLR1	Triacyl lipopeptides	Lipopeptide	Bacterial (lipoproteins)	MyD88 -> NF-kB -> inflammatory mediators
TLR2	Glycolipids	Glycolipid	Bacterial (peptidoglycan-associated)	MyD88 -> NF-kB -> inflammatory mediators
	Lipopeptides and proteolipids	Lipopeptide/Proteolipid	Bacterial (peptidoglycan-associated)	
	Lipoteichoic acid	Glycophospholipid	Gram-positive bacteria	
	Biglycan	Proteoglycan	Host cells	
	Versican	Proteoglycan	Host cells	
	Hyaluronan	Glycosaminoglycan	Host cells (extracellular matrix)	
	Heat-shock protein 70 (HSP70)	Protein	Host cells	
	Zymosan (β-glucan)	Polysaccharide (β-glucan)	Fungi	
	HMGB1	Protein	Host cells	
TLR3	Double-stranded RNA (dsRNA)	Nucleic acid (dsRNA)	Viral	TRIF -> NF-kB -> inflammatory mediators
	Polyinosinic-polycytidylic acid (poly I:C)	Synthetic dsRNA analog	Experimental/viral mimic	
	Lipopolysaccharide (LPS)	Glycolipid	Gram-negative bacteria	
	Heat-shock proteins (various)	Protein	Bacterial and host cells	
	RNA	Nucleic acid	Host cells	
	Fibrinogen	Protein	Host cells (extracellular matrix)	
TLR4	Lipopolysaccharide (LPS)	Glycolipid	Gram-negative bacteria	MyD88 -> NF-kB -> inflammatory mediators TRIF -> NF-kB -> inflammatory mediators
	Biglycan	Proteoglycan	Host cells	
	Heparan sulfate fragments	Glycosaminoglycan	Host cells (extracellular matrix)	
	Hyaluronan	Glycosaminoglycan	Host cells (extracellular matrix)	
	Heat-shock protein	Protein	Host cells	
	Nickel (Ni^2+^)	Metal ion	Environmental/exogenous metal	
	HMGB1	Protein	Host cells	
TLR5	Flagellin	Protein	Bacterial (flagellated species)	MyD88 -> NF-kB -> inflammatory mediators
	Profilin (Toxoplasma gondii)	Protein	Protozoan (T. gondii)	
TLR6	Diacyl lipopeptides	Lipopeptide	Mycoplasma (bacterial lipoproteins)	MyD88 -> NF-kB -> inflammatory mediators
TLR7	Imidazoquinoline derivatives	Small synthetic compound	Synthetic	MyD88 -> NF-kB -> inflammatory mediators
	Loxoribine (guanosine analog)	Nucleoside analog	Synthetic	
	Bropirimine	Small synthetic compound	Synthetic	
	Resiquimod	Small synthetic compound	Synthetic	
	Single-stranded RNA (ssRNA)	Nucleic acid (ssRNA)	RNA viruses	
TLR8	Small synthetic compounds	Small organic molecules	Synthetic	MyD88 -> NF-kB -> inflammatory mediators
	Single-stranded viral RNA	Nucleic acid (ssRNA)	RNA viruses	
	Phagocytosed bacterial RNA	Nucleic acid (ssRNA)	Bacteria (intracellular)	
TLR9	Unmethylated CpG–oligodeoxynucleotides (CpG DNA)	Nucleic acid (DNA)	Bacteria, DNA viruses	MyD88 -> NF-kB -> inflammatory mediators
	DNA (mitochondrial)	Nucleic acid	Host cells	
	DNA	Nucleic acid	Host cells	
	HMGB1	Protein	Host cells	
TLR11	Profilin (Toxoplasma gondii)	Protein	Protozoan (T. gondii)	MyD88 -> NF-kB -> inflammatory mediators
TLR12	Profilin (Toxoplasma gondii)	Protein	Protozoan (T. gondii)	MyD88 -> NF-kB -> inflammatory mediators
TLR13	Bacterial ribosomal RNA sequences (e.g., 23S rRNA)	Nucleic acid (rRNA)	Bacteria	MyD88 -> NF-kB -> inflammatory mediators

**Table 2:** Summary of Toll-like receptor (TLR) involvement in metabolic regulation, cognitive function (memory), and emotional behavior across preclinical and clinical studies. The table compiles evidence for the role of individual TLRs in metabolic alterations (e.g., insulin resistance, glucose metabolism, adiposity), cognition and memory (e.g., spatial memory, learning, synaptic plasticity), and emotional regulation (e.g., depression-, anxiety-, and stress-related behaviors). Data includes results from human studies, rodent knockout (KO) models, pharmacological interventions, and gene expression analyses. Arrows indicate direction of change (↑ increase, ↓ decrease) of expression levels, and (+)/(−) symbols represent pharmacological or genetic activation or inhibition, respectively. KO refers to knockout models. References are indicated in brackets.

	Metabolism Alteration	Cognition—Memory	Emotional Regulation
TLR1			below normal levels with antidepressant treatment [41]
TLR2	(+) insulin resistance [48] (+) increased production of kynurenine [49] ↑ in obesity [50] ↓ with HFD [51,52] KO protected from insulin resistance [53,54,55] KO higher BW and reduced food intake [56] KO exhibit a significant reduction in adipocyte size in epididymal fat under low-fat diet [57]	(+) impaired spatial memory and learning [48,58] (+) fear memory destabilization [59] (+) postnatally impairs learning and memory in the adulthood [60] (−) improving learning and memory [61] KO prevents surgery induced memory impairment and fear conditioning [62] KO slower learning speed [63] KO impaired cognitive function [64,65]	(+) necessary in social defeat stress [66,67] ↑ in depression and suicidal behavior [42,43,44,45,68] ↑ in MDD patients with comorbid multiple sclerosis [47] ↑ in poor omega-3 diet depressed female rats [49] (−) attenuate the obesity-associated depressive-like behavior [50] (−)improving anxiety-like behavior [61] below normal levels with antidepressant treatment [41] KO increased anxiety-like state [64] KO increased depression-like state [69]
TLR3	Astroglial metabolism alteration [70] ↓ in lymphocytes and adipocytes linked to metabolic inflammation [71]	(+) impaired memory and learning [72,73] (+) reduces apical dendritic spine density [74,75] (+) induces neuronal apoptosis [76] (−) improve chronic constriction injury memory impairment [77] (−) enhanced hippocampus dependent learning and memory [78] (−) impairs amygdala-dependent learning and memory [78] (−) improves cognitive decline induced by chronic neuropathic pain [77] KO impaired spatial but not working memory [79]	↑ in resistant depression patients [41] ↑ in depression and suicidal behavior [41,43,80,81] (+) inhibition of neuronal plasticity in vitro [76] recover normal levels with antidepressant treatment [41] (−) attenuate anxiety-like behavior in male [82] (−) attenuate anxiety-like behavior in female [83] KO reduce anxiety-like behavior [78,79]
TLR4	(+) increased production of kynurenine [49] (+) increases hippocampal energy metabolism and p-AMPK levels [84] ↑ in type 1 and 2 diabetes [85,86,87] ↑ in placenta in models of gestational diabetes mellitus [88] (−) prevents associated affective disturbances induced by a high cholesterol diet [89] KO higher BW and reduced food intake [56] KO exhibit a significant reduction in adipocyte size in epididymal fat under low-fat diet [57]	(+) impaired spatial memory and learning [87,90] (+) fear memory destabilization [59] ↑ neonatally leads to memory deficits in adulthood [91] (−) improving learning and memory [61,92] (−) alters the regulation of spatial reference memory and fear learning [93] (−) protects against alcohol synaptic plasticity and cognitive function impairment [90,94]	(+) necessary in social defeat stress [66,67] (+) in anxiety-like diabetic mice [86] (+) susceptibility to depression-like behavior [41,81] (+) necessary in social defeat stress [66,67] ↑ in social stressed mice [95,96] ↑ in depressed diabetic mice [86] ↑ in depression and suicidal behavior [97] ↑ in poor omega-3 diet depressed female rats [49] ↑ in chronic mild stress and recovered by antidepressants [84] (−) therapeutic role for TLR4 blockade in stress-related neuropsychiatric disorders [98] ↓ exerts an anti-depressive action [99,100,101] ↓ with melatonin treatment [92] below normal levels with antidepressant treatment [41] KO protected against persistent depression in female mice [102] KO decreases anxiety-like behavior in males [103] KO anxiety-like phenotype [104] KO enhances stress-induced responses in females [105] KO protected against corticotropin-releasing factor release induced by stress [106]
TLR5	↑ in type 2 diabetes [107] ↑ in obesity related dysbiosis [108] ↑ by intense exercise [109] ↑ in placenta in models of gestational diabetes mellitus [88] KO glucose intolerance/insulin resistance [110,111,112,113,114,115] KO mice increase adiposity [110,112,113,114,115] KO mice females protected from obesity [112] KO humans protect from weight gain [110] KO exhibit a significant reduction in adipocyte size in epididymal fat under low-fat diet [57] KO do not exhibit metabolic abnormalities [116,117]		↑ in depression and normalized with antidepressant treatment [41,81] KO reduce anxiety behavior [118]
TLR6	↑ in non-alcoholic fatty liver patients [119] ↓ with HFD [52]		↓ below normal levels with antidepressant treatment [41]
TLR7	(+) exacerbates HFD-induced dysregulation of glucose [120,121] (+) increases glycolysis via the AKT-mTOR-IRF4 axis [122] KO reduced BW gain with HFD [123]	(+) enhanced fear memory [124] (−) improved hippocampus-dependent memory [125] KO enhanced fear memory after rotarod [126] KO impairs fear memory [127] KO enhanced spatial memory after rotarod [126]	↑ in depression and normalized with antidepressant treatment [41,81] ↑ in gestation lead to anxiety-like behavior in adulthood [128,129] (−) helps in models of postpartum depression [130] KO reduce anxiety behavior [101] KO anxiety-like behavior [86] KO blocks chronic stress-induced immune suppression [87]
TLR8	(+) inhibits glucose uptake and glycolysis in human [110,111]	(+) inhibits neurite outgrowth and induce apoptosis in vitro [112]	Particular DNA methylation in TLR8 gene in PTSD related with childhood abuse [57]; refuted in [113]
TLR9	(+) activates AMPK [131,132] ↑ in diabetes condition [133] KO deficient AMPK and GLUT4 activation [132]	(+) leads to cognitive improvements [134] (+) CpG DNA mediated impaired spatial memory [135] KO impaired working memory [79,136] KO impaired fear memory [136]	(+) prevents post-traumatic consequences in stressed mice [137] (+) depressive- and anxiety-like behaviors induced by CUMS [138] ↑ in depression and normalized with antidepressant treatment [41] (−) attenuates stress-induced social behavior deficits [139] KO shows hyperactivity [79] KO resistant to stress-induced immune suppression [140]
TLR10	↑ in type 2 diabetes [141] (−) higher blood glucose and lower insulin levels [142] (−) obesity resistance [142]		
TLR11/12	↑ in obesity-associated inflammation [143] ↓ by quinoa while upregulating lipid metabolism [144]		

**Table 3:** Summary of main effects of TLRs in animal models and human studies. Arrows indicate direction of change (↑ increase, ↓ decrease) of expression levels, and (+)/(−) symbols represent pharmacological or genetic activation or inhibition, respectively.

TLR	Human	Murine (Mouse/Rat)
TLR1	Below normal levels with antidepressant treatment	
TLR2	↑ in obesity, MDD and IBS	Reduction protects against HFD-induced insulin resistance and surgery-induced memory deficits; ↓ anxiety-/depression-like behavior
TLR3	↑ in depression	(+) impaired memmory and increased depressive and anxiety behavior. (−) improves memmory and shows reduced anxiety
TLR4	↑ in obesity, diabetes and depression	(+) impaired memmory and increased depressive and anxiety behavior. (−) improves memmory and shows reduced anxiety and depression
TLR5	↑ in obesity, diabetes and depression	↑ in diabetes, ↓with HFD. (−) glucose intolerance, increassed adiposity but ptrotected from obesity
TLR6	↑ in non-alcoholic fatty liver patients	↓ with HFD
TLR7	↓ in obese elderly men and ↑in depression	(+)dysregulation of glucose, enhanced fear memory and anxiety (−) enhanced spatial memory, reduce anxiety and depression sintoms
TLR8	(+) inhibits glucose uptake and glycolysis	
TLR9	↑ diabetes and depression	(+) activates AMPK, cognitive improvement and depression and anxiety-like behaviors. (−) deficent in AMPK and GLUT4, impaired memory and reduced stress
TLR10	↑ type 2 diabetes (−) higher blood glucose, lower insulin levels and obesity resistance	
TLR11–12		↑ obessity associated inflammation

## Data Availability

No new data were created or analyzed in this study.

## References

[1] Marshall J.S., Warrington R., Watson W., Kim H.L. (2018). An introduction to immunology and immunopathology. Allergy Asthma Clin. Immunol..

[2] Medina K.L. (2016). Overview of the immune system. Handb. Clin. Neurol..

[3] Weavers H., Martin P. (2020). The cell biology of inflammation: From common traits to remarkable immunological adaptations. J. Cell Biol..

[4] Schupbach T., Wieschaus E. (1989). Female Sterile Mutations on the Second Chromosome of Drosophila melanogaster. I. Maternal Effect Mutations. Genetics.

[5] Anderson K.V., Nüsslein-Volhard C. (1984). Information for the dorsal–ventral pattern of the Drosophila embryo is stored as maternal mRNA. Nature.

[6] Lemaitre B., Nicolas E., Michaut L. (1996). The Dorsoventral Regulatory Gene Cassette spä tzle/Toll/cactus Controls the Potent Antifungal Response in Drosophila Adults. Cell.

[7] Poltorak A., He X., Smirnova I., Liu M.-Y., Van Huffel C., Du X., Birdwell D., Alejos E., Silva M., Galanos C. (1998). Defective LPS Signaling in C3H/HeJ and C57BL/10ScCr Mice: Mutations in Tlr4 Gene. Science.

[8] Lemaitre B., Hoffmann J. (2007). The Host Defense of *Drosophila melanogaster*. Annu. Rev. Immunol..

[9] Nüsslein-Volhard C. (2022). The Toll gene in Drosophila pattern formation. Trends Genet..

[10] Liu G., Zhao Y. (2007). Toll-like receptors and immune regulation: Their direct and indirect modulation on regulatory CD4^+^ CD25^+^ T cells. Immunology.

[11] Amarante-Mendes G.P., Adjemian S., Branco L.M., Zanetti L.C., Weinlich R., Bortoluci K.R. (2018). Pattern recognition receptors and the host cell death molecular machinery. Front. Immunol..

[12] Chen L., Deng H., Cui H., Fang J., Zuo Z., Deng J., Li Y., Wang X., Zhao L. (2017). Oncotarget 1 Inflammatory Responses and Inflammation-Associated Diseases in Organs. https://www.oncotarget.com/article/23208/text/.

[13] de Heredia F.P., Gómez-Martínez S., Marcos A. (2012). Obesity, inflammation and the immune system. Proc. Nutr. Soc..

[14] Parkin J., Cohen B. (2001). An overview of the immune system. Lancet.

[15] Tsai S.-Y., Gildengers A.G., Hsu J.-L., Chung K.-H., Chen P.-H., Huang Y.-J. (2019). Inflammation associated with volume reduction in the gray matter and hippocampus of older patients with bipolar disorder. J. Affect. Disord..

[16] Mahajan G.J., Vallender E.J., Garrett M.R., Challagundla L., Overholser J.C., Jurjus G., Dieter L., Syed M., Romero D.G., Benghuzzi H. (2018). Altered neuro-inflammatory gene expression in hippocampus in major depressive disorder. Prog. Neuro-Psychopharmacol. Biol. Psychiatry.

[17] Cortés M., Brischetto A., Martinez-Campanario M.C., Ninfali C., Domínguez V., Fernández S., Celis R., Esteve-Codina A., Lozano J.J., Sidorova J. (2023). Inflammatory macrophages reprogram to immunosuppression by reducing mitochondrial translation. Nat. Commun..

[18] van de Vyver M. (2023). Immunology of chronic low-grade inflammation: Relationship with metabolic function. J. Endocrinol..

[19] Hu T., Liu C.-H., Lei M., Zeng Q., Li L., Tang H., Zhang N. (2024). Metabolic regulation of the immune system in health and diseases: Mechanisms and interventions. Signal Transduct. Target. Ther..

[20] Penninx B.W., Lamers F., Jansen R., Berk M., Khandaker G.M., De Picker L., Milaneschi Y. (2025). Immuno-metabolic depression: From concept to implementation. Lancet Reg. Health-Eur..

[21] Jayatissa M.N., Bisgaard C., Tingström A., Papp M., Wiborg O. (2006). Hippocampal cytogenesis correlates to escitalopram-mediated recovery in a chronic mild stress rat model of depression. Neuropsychopharmacology.

[22] Xi G., Hui J., Zhang Z., Liu S., Zhang X., Teng G., Chan K.C., Wu E.X., Nie B., Shan B. (2011). Learning and memory alterations are associated with hippocampal N-acetylaspartate in a rat model of depression as measured by 1H-MRS. PLoS ONE.

[23] Zhao J., Jiang W., Wang X., Cai Z., Liu Z., Liu G. (2020). Exercise, brain plasticity, and depression. CNS Neurosci. Ther..

[24] Mahati K., Bhagya V., Christofer T., Sneha A., Rao B.S. (2016). Enriched environment ameliorates depression-induced cognitive deficits and restores abnormal hippocampal synaptic plasticity. Neurobiol. Learn. Mem..

[25] Garcia-Castillo V., Komatsu R., Clua P., Indo Y., Takagi M., Salva S., Islam A., Alvarez S., Takahashi H., Garcia-Cancino A. (2019). Evaluation of the Immunomodulatory Activities of the Probiotic Strain Lactobacillus fermentum UCO-979C. Front. Immunol..

[26] Llewellyn A., Foey A. (2017). Probiotic Modulation of Innate Cell Pathogen Sensing and Signaling Events. Nutrients.

[27] Kawai T., Akira S. (2006). TLR signaling. Cell Death Differ..

[28] Takeuchi O., Hoshino K., Kawai T., Sanjo H., Takada H., Ogawa T., Takeda K., Akira S. (1999). Differential Roles of TLR2 and TLR4 in Recognition of Gram-Negative and Gram-Positive Bacterial Cell Wall Components. Immunity.

[29] Hayashi F., Smith K.D., Ozinsky A., Hawn T.R., Yi E.C., Goodlett D.R., Eng J.K., Akira S., Underhill D.M., Aderem A. (2001). The innate immune response to bacterial ¯agellin ismediated by Toll-like receptor 5. Nature.

[30] Brightbill H.D., Libraty D.H., Krutzik S.R., Yang R.-B., Belisle J.T., Bleharski J.R., Maitland M., Norgard M.V., Plevy S.E., Smale S.T. (1999). Host Defense Mechanisms Triggered by Microbial Lipoproteins Through Toll-Like Receptors. Science.

[31] Takeda K., Kaisho T., Akira S. (2003). Toll-like receptors. Annu. Rev. Immunol..

[32] Kawai T., Akira S. (2010). The role of pattern-recognition receptors in innate immunity: Update on Toll-like receptors. Nat. Immunol..

[33] Guo H., Callaway J.B., Ting J.P.-Y. (2015). Inflammasomes: Mechanism of action, role in disease, and therapeutics. Nat. Med..

[34] Hanamsagar R., Hanke M.L., Kielian T. (2012). Toll-like receptor (TLR) and inflammasome actions in the central nervous system. Trends Immunol..

[35] Okun E., Griffioen K.J., Mattson M.P. (2011). Toll-like receptor signaling in neural plasticity and disease. Trends Neurosci..

[36] Kaul D., Habbel P., Derkow K., Krüger C., Franzoni E., Wulczyn F.G., Bereswill S., Nitsch R., Schott E., Veh R. (2012). Expression of toll-like receptors in the developing brain. PLoS ONE.

[37] Barak B., Feldman N., Okun E. (2014). Toll-like receptors as developmental tools that regulate neurogenesis during development: An update. Front. Neurosci..

[38] Hanke M.L., Kielian T. (2011). Toll-like receptors in health and disease in the brain: Mechanisms and therapeutic potential. Clin. Sci..

[39] Frederiksen H.R., Haukedal H., Freude K. (2019). Cell type specific expression of toll-like receptors in human brains and implications in Alzheimer’s disease. BioMed Res. Int..

[40] Nishimura M., Naito S. (2008). Tissue-specific mRNA Expression Profiles of Human Solute Carrier Transporter Superfamilies. Drug Metab. Pharmacokinet..

[41] Hung Y.-Y., Huang K.-W., Kang H.-Y., Huang G.Y.-L., Huang T.-L. (2016). Antidepressants normalize elevated Toll-like receptor profile in major depressive disorder. Psychopharmacology.

[42] Wu S., Jiang Q., Wang J., Wu D., Ren Y. (2024). Immune-related gene characterization and biological mechanisms in major depressive disorder revealed based on transcriptomics and network pharmacology. Front. Psychiatry.

[43] Pandey G.N., Rizavi H.S., Bhaumik R., Ren X. (2019). Innate immunity in the postmortem brain of depressed and suicide subjects: Role of Toll-like receptors. Brain Behav. Immun..

[44] Zeng D., He S., Ma C., Wen Y., Song W., Xu Q., Zhao N., Wang Q., Yu Y., Shen Y. (2020). Network-based approach to identify molecular signatures in the brains of depressed suicides. Psychiatry Res..

[45] Hung Y.-Y. (2018). Antidepressants Improve Negative Regulation of Toll-Like Receptor Signaling in Monocytes from Patients with Major Depression. Neuroimmunomodulation.

[46] Wieck A., Grassi-Oliveira R., Prado C.H.D., Viola T.W., Petersen L.E., Porto B., Teixeira A.L., Bauer M.E. (2016). Toll-like receptor expression and function in type I bipolar disorder. Brain Behav. Immun..

[47] Sales M.C., Kasahara T.M., Sacramento P.M., Rossi Á.D., Cafasso M.O.S., Oyamada H.A., Hygino J., Alvim F., Andrade R.M., Vasconcelos C.C. (2020). Selective serotonin reuptake inhibitor attenuates the hyperresponsiveness of TLR2^+^ and TLR4^+^ Th17/Tc17-like cells in multiple sclerosis patients with major depression. Immunology.

[48] Ahuja S., Uniyal A., Akhtar A., Sah S.P. (2019). Alpha lipoic acid and metformin alleviates experimentally induced insulin resistance and cognitive deficit by modulation of TLR2 signalling. Pharmacol. Rep..

[49] Morgese M.G., Schiavone S., Maffione A.B., Tucci P., Trabace L. (2020). Depressive-like phenotype evoked by lifelong nutritional omega-3 deficiency in female rats: Crosstalk among kynurenine, Toll-like receptors and amyloid beta oligomers. Brain Behav. Immun..

[50] Agusti A., Moya-Pérez A., Campillo I., la Paz S.M.-D., Cerrudo V., Perez-Villalba A., Sanz Y. (2018). Bifidobacterium pseudocatenulatum CECT 7765 Ameliorates Neuroendocrine Alterations Associated with an Exaggerated Stress Response and Anhedonia in Obese Mice. Mol. Neurobiol..

[51] Dutheil S., Ota K.T., Wohleb E.S., Rasmussen K., Duman R.S. (2016). High-Fat Diet Induced Anxiety and Anhedonia: Impact on Brain Homeostasis and Inflammation. Neuropsychopharmacology.

[52] Betanzos-Cabrera D.E.-L.G. (2012). El tejido adiposo y el tejido hepático de los ratones alimentados con una dieta alta en grasa tienen un decremento en la expresión del mRNA del ‘toll like receptor’ (TLR)2 y del TLR6. Nutr. Hosp..

[53] Himes R.W., Smith C.W. (2010). *Tlr2* is critical for diet-induced metabolic syndrome in a murine model. FASEB J..

[54] Kuo L.-H., Tsai P.-J., Jiang M.-J., Chuang Y.-L., Yu L., Lai K.-T.A., Tsai Y.-S. (2011). Toll-like receptor 2 deficiency improves insulin sensitivity and hepatic insulin signalling in the mouse. Diabetologia.

[55] Ehses J.A., Meier D.T., Wueest S., Rytka J., Boller S., Wielinga P.Y., Schraenen A., Lemaire K., Debray S., Van Lommel L. (2010). Toll-like receptor 2-deficient mice are protected from insulin resistance and beta cell dysfunction induced by a high-fat diet. Diabetologia.

[56] Okun E., Griffioen K.J., Rothman S., Wan R., Cong W.-N., De Cabo R., Martin-Montalvo A., Levette A., Maudsley S., Martin B. (2013). Toll-like receptors 2 and 4 modulate autonomic control of heart rate and energy metabolism. Brain Behav. Immun..

[57] Rau C.-S., Wu S.-C., Lu T.-H., Wu Y.-C., Wu C.-J., Chien P.-C., Kuo P.-J., Lin C.-W., Tsai C.-W., Hsieh C.-H. (2018). Effect of low-fat diet in obese mice lacking toll-like receptors. Nutrients.

[58] Pang X., Zhang P., Zhou Y., Zhao J., Liu H. (2020). Dexmedetomidine pretreatment attenuates isoflurane-induced neurotoxicity via inhibiting the TLR2/NF-κB signaling pathway in neonatal rats. Exp. Mol. Pathol..

[59] Chen R., Wang Z., Lin Q., Hou X., Jiang Y., Le Q., Liu X., Ma L., Wang F. (2024). Destabilization of fear memory by Rac1-driven engram-microglia communication in hippocampus. Brain Behav. Immun..

[60] Madar R., Rotter A., Ben-Asher H.W., Mughal M.R., Arumugam T.V., Wood W., Becker K., Mattson M.P., Okun E. (2015). Postnatal TLR2 activation impairs learning and memory in adulthood. Brain Behav. Immun..

[61] Mohseni-Moghaddam P., Dogani M., Hatami M., Roohollahi S., Amiresmaeli A., Askari N. (2022). A behavioral and molecular study; ameliorated anxiety-like behavior and cognitive dysfunction in a rat model of chronic unpredictable stress treated with oregano extract. Brain Behav..

[62] Lin F., Shan W., Zheng Y., Pan L., Zuo Z. (2021). Toll-like receptor 2 activation and up-regulation by high mobility group box-1 contribute to post-operative neuroinflammation and cognitive dysfunction in mice. J. Neurochem..

[63] Bae H.J., Kim J., Bae H.J., Park K., Yang X., Cho Y.-J., Jung S.Y., Park S.J., Ryu J.H. (2022). Effects of repetitive training on learning and memory performance of TLR2 KO mice. Behav. Brain Res..

[64] Hu Y., Sun X., Wang S., Zhou C., Lin L., Ding X., Han J., Zhou Y., Jin G., Wang Y. (2021). Toll-like receptor-2 gene knockout results in neurobehavioral dysfunctions and multiple brain structural and functional abnormalities in mice. Brain Behav. Immun..

[65] Park S.J., Lee J.Y., Kim S.J., Choi S.-Y., Yune T.Y., Ryu J.H. (2015). Toll-like receptor-2 deficiency induces schizophrenia-like behaviors in mice. Sci. Rep..

[66] Nie X., Kitaoka S., Tanaka K., Segi-Nishida E., Imoto Y., Ogawa A., Nakano F., Tomohiro A., Nakayama K., Taniguchi M. (2018). The Innate Immune Receptors TLR2/4 Mediate Repeated Social Defeat Stress-Induced Social Avoidance through Prefrontal Microglial Activation. Neuron.

[67] Kitaoka S., Tomohiro A., Ukeshima S., Liu K., Wake H., Kimura S.H., Yamamoto Y., Nishibori M., Furuyashiki T. (2023). Repeated Social Defeat Stress Induces HMGB1 Nuclear Export in Prefrontal Neurons, Leading to Social Avoidance in Mice. Cells.

[68] Giridharan V.V., Réus G.Z., Selvaraj S., Scaini G., Barichello T., Quevedo J. (2019). Maternal deprivation increases microglial activation and neuroinflammatory markers in the prefrontal cortex and hippocampus of infant rats. J. Psychiatr. Res..

[69] Medina-Rodriguez E.M., Cheng Y., Michalek S.M., Beurel E., Jope R.S. (2020). Toll-like receptor 2 (TLR2)-deficiency impairs male mouse recovery from a depression-like state. Brain Behav. Immun..

[70] Salmina A.B., Komleva Y.K., Lopatina O.L., Kuvacheva N.V., Gorina Y.V., Panina Y.A., Uspenskaya Y.A., Petrova M.M., Demko I.V., Zamay A.S. (2015). Astroglial control of neuroinflammation: TLR3-mediated dsRNA-sensing pathways are in the focus. Prog. Neurobiol..

[71] Latorre J., Moreno-Navarrete J.M., Sabater M., Buxo M., Rodriguez-Hermosa J.I., Girones J., Fort J.M., Vilallonga R., Ricart W., Simo R. (2018). Decreased TLR3 in Hyperplastic Adipose Tissue, Blood and Inflamed Adipocytes is Related to Metabolic Inflammation. Cell Physiol. Biochem..

[72] Viola T.W., Creutzberg K.C., Zaparte A., Kestering-Ferreira É., Tractenberg S.G., Centeno-Silva A., Orso R., Lumertz F.S., Brietzke E., Wearick-Silva L.E. (2019). Acute neuroinflammation elicited by TLR-3 systemic activation combined with early life stress induces working memory impairments in male adolescent mice. Behav. Brain Res..

[73] Baghel M.S., Singh B., Dhuriya Y.K., Shukla R.K., Patro N., Khanna V.K., Patro I.K., Thakur M.K. (2018). Postnatal exposure to poly (I:C) impairs learning and memory through changes in synaptic plasticity gene expression in developing rat brain. Neurobiol. Learn. Mem..

[74] Sanchez-Mendoza E.H., Camblor-Perujo S., Nascentes-Melo L.M., Dzyubenko E., Fleischer M., de Carvalho T.S., Schmitt L.-I., Leo M., Hagenacker T., Herring A. (2020). Compromised Hippocampal Neuroplasticity in the Interferon-α and Toll-like Receptor-3 Activation-Induced Mouse Depression Model. Mol. Neurobiol..

[75] Hoyo-Becerra C., Liu Z., Yao J., Kaltwasser B., Gerken G., Hermann D.M., Schlaak J.F. (2014). Rapid Regulation of Depression-Associated Genes in a New Mouse Model Mimicking Interferon-α-Related Depression in Hepatitis C Virus Infection. Mol. Neurobiol..

[76] Hoyo-Becerra C., Huebener A., Trippler M., Lutterbeck M., Liu Z.J., Truebner K., Bajanowski T., Gerken G., Hermann D.M., Schlaak J.F. (2013). Concomitant interferon alpha stimulation and TLR3 activation induces neuronal expression of depression-related genes that are elevated in the brain of suicidal persons. PLoS ONE.

[77] Zhang X., Gao R., Zhang C., Teng Y., Chen H., Li Q., Liu C., Wu J., Wei L., Deng L. (2023). Extracellular RNAs-TLR3 signaling contributes to cognitive impairment after chronic neuropathic pain in mice. Signal Transduct. Target. Ther..

[78] Okun E., Griffioen K., Barak B., Roberts N.J., Castro K., Pita M.A., Cheng A., Mughal M.R., Wan R., Ashery U. (2010). Toll-like receptor 3 inhibits memory retention and constrains adult hippocampal neurogenesis. Proc. Natl. Acad. Sci. USA.

[79] Vargas-Calderón H., Ortega-Robles E., Rocha L., Yu P., Arias-Carrión O. (2024). Motor, Cognitive, and Behavioral Impairment in TLR3 and TLR9 Deficient Male Mice: Insights into the Non-Immunological Roles of Toll-Like Receptors. Arch. Med. Res..

[80] Pandey G.N., Rizavi H.S., Ren X., Bhaumik R., Dwivedi Y. (2014). Toll-like receptors in the depressed and suicide brain. J. Psychiatr. Res..

[81] Hung Y.-Y., Kang H.-Y., Huang K.-W., Huang T.-L. (2014). Association between toll-like receptors expression and major depressive disorder. Psychiatry Res..

[82] Wang X., Yu H., Wang C., Liu Y., You J., Wang P., Xu G., Shen H., Yao H., Lan X. (2020). Chronic ethanol exposure induces neuroinflammation in H4 cells through TLR3 / NF-κB pathway and anxiety-like behavior in male C57BL/6 mice. Toxicology.

[83] Flannery L.E., Kerr D.M., Finn D.P., Roche M. (2018). FAAH inhibition attenuates TLR3-mediated hyperthermia, nociceptive- and anxiety-like behaviour in female rats. Behav. Brain Res..

[84] Khedr L., Nassar N., Rashed L., El-Denshary E., Abdel-Tawab A. (2019). TLR4 signaling modulation of PGC1-α mediated mitochondrial biogenesis in the LPS-Chronic mild stress model: Effect of fluoxetine and pentoxiyfylline. Life Sci..

[85] Novoselova E.G., Glushkova O.V., Lunin S.M., Khrenov M.O., Novoselova T.V., Parfenyuk S.B., Fesenko E.E. (2016). Signaling, stress response and apoptosis in pre-diabetes and diabetes: Restoring immune balance in mice with alloxan-induced type 1 diabetes mellitus. Int. Immunopharmacol..

[86] Habib M., Shaker S., El-Gayar N., Aboul-Fotouh S., Resstel L.B.M. (2015). The effects of antidepressants “fluoxetine and imipramine” on vascular abnormalities and toll like receptor-4 expression in diabetic and non-diabetic rats exposed to chronic stress. PLoS ONE.

[87] Kawamoto E.M., Cutler R.G., Rothman S.M., Mattson M.P., Camandola S. (2014). TLR4-dependent metabolic changes are associated with cognitive impairment in an animal model of type 1 diabetes. Biochem. Biophys. Res. Commun..

[88] Liu J., Chen Y., Laurent I., Yang P., Xiao X., Li X. (2024). Gestational diabetes exacerbates intrauterine microbial exposure induced intestinal microbiota change in offspring contributing to increased immune response. Nutr. Diabetes.

[89] Strekalova T., Costa-Nunes J.P., Veniaminova E., Kubatiev A., Lesch K.-P., Chekhonin V.P., Evans M.C., Steinbusch H.W. (2016). Insulin receptor sensitizer, dicholine succinate, prevents both Toll-like receptor 4 (TLR4) upregulation and affective changes induced by a high-cholesterol diet in mice. J. Affect. Disord..

[90] Deschamps C., Uyttersprot F., Debris M., Marié C., Fouquet G., Marcq I., Vilpoux C., Naassila M., Pierrefiche O. (2022). Anti-inflammatory drugs prevent memory and hippocampal plasticity deficits following initial binge-like alcohol exposure in adolescent male rats. Psychopharmacology.

[91] Zaniani N.R., Roohbakhsh A., Moghimi A., Mehri S. (2022). Protective effect of Toll-like receptor 4 antagonist on inflammation, EEG, and memory changes following febrile seizure in Wistar rats. Behav. Brain Res..

[92] Cui Y., Yang M., Wang Y., Ren J., Lin P., Cui C., Song J., He Q., Hu H., Wang K. (2021). Melatonin prevents diabetes-associated cognitive dysfunction from microglia-mediated neuroinflammation by activating autophagy via TLR4/Akt/mTOR pathway. FASEB J..

[93] Okun E., Barak B., Saada-Madar R., Rothman S.M., Griffioen K.J., Roberts N., Castro K., Mughal M.R., Pita M.A., Stranahan A.M. (2012). Evidence for a Developmental Role for TLR4 in Learning and Memory. PLoS ONE.

[94] Vetreno R.P., Lawrimore C.J., Rowsey P.J., Crews F.T. (2018). Persistent adult neuroimmune activation and loss of hippocampal neurogenesis following adolescent ethanol exposure: Blockade by exercise and the anti-inflammatory drug indomethacin. Front. Neurosci..

[95] Motooka Y., Shinohara R., Kitaoka S., Uryu A., Li D., Neyama H., Cui Y., Kida T., Arakaki W., Doi H. (2025). Alteration of COX-1 and TLR4 expression in the mouse brain during chronic social defeat stress revealed by Positron Emission Tomography study. J. Pharmacol. Sci..

[96] Gárate I., Bueno B.G., Madrigal J., Caso J., Alou L., Gomez-Lus M.L., Micó J.A., Leza J.C. (2013). Stress-induced neuroinflammation: Role of the toll-like receptor-4 pathway. Biol. Psychiatry.

[97] Wang J., Yang C., Liu Z., Li X., Liu M., Wang Y., Zhang K., Sun N. (2020). Association of the TLR4 gene with depressive symptoms and antidepressant efficacy in major depressive disorder. Neurosci. Lett..

[98] Gárate I., García-Bueno B., Madrigal J.L.M., Caso J.R., Alou L., Gómez-Lus M.L., Leza J.C. (2014). Toll-like 4 receptor inhibitor TAK-242 decreases neuroinflammation in rat brain frontal cortex after stress. J. Neuroinflammation.

[99] Zhang K., Lin W., Zhang J., Zhao Y., Wang X., Zhao M. (2020). Effect of Toll-like receptor 4 on depressive-like behaviors induced by chronic social defeat stress. Brain Behav..

[100] Hu L., Zeng X., Yang K., Peng H., Chen J. (2022). n-3 polyunsaturated fatty acids improve depression-like behavior by inhibiting hippocampal neuroinflammation in mice via reducing TLR4 expression. Immun. Inflamm. Dis..

[101] Huang X., Fei G.-Q., Liu W.-J., Ding J., Wang Y., Wang H., Ji J.-L., Wang X. (2020). Adipose-derived mesenchymal stem cells protect against CMS-induced depression-like behaviors in mice via regulating the Nrf2/HO-1 and TLR4/NF-κB signaling pathways. Acta Pharmacol. Sin..

[102] Yang E.-J., Frolinger T., Iqbal U., Estill M., Shen L., Trageser K.J., Pasinetti G.M. (2024). The role of the Toll like receptor 4 signaling in sex-specific persistency of depression-like behavior in response to chronic stress. Brain Behav. Immun..

[103] Li Y., Zhu S., Xie K., Feng X., Chen L., Wu X., Sun Z., Shu G., Wang S., Zhu C. (2022). TLR4 in Tph2 neurons modulates anxiety-related behaviors in a sex-dependent manner. Neuropharmacology.

[104] Femenia T., Qian Y., Arentsen T., Forssberg H., Heijtz R.D. (2018). Toll-like receptor-4 regulates anxiety-like behavior and DARPP-32 phosphorylation. Brain Behav. Immun..

[105] Quave C.B., Nieto S.J., Haile C.N., Kosten T.A. (2021). Immune receptor toll-like receptor 4 contributes to stress-induced affective responses in a sex-specific manner. Brain Behav. Immun.-Health.

[106] Varodayan F.P., Khom S., Patel R.R., Steinman M.Q., Hedges D.M., Oleata C.S., Homanics G.E., Roberto M., Bajo M. (2018). Role of TLR4 in the Modulation of Central Amygdala GABA Transmission by CRF Following Restraint Stress. Alcohol Alcohol..

[107] Scheithauer T.P., Herrema H., Yu H., Bakker G.J., Winkelmeijer M., Soukhatcheva G., Dai D., Ma C., Havik S.R., Balvers M. (2022). Gut-derived bacterial flagellin induces beta-cell inflammation and dysfunction. Gut Microbes.

[108] Pekkala S., Munukka E., Kong L., Pöllänen E., Autio R., Roos C., Wiklund P., Fischer-Posovszky P., Wabitsch M., Alen M. (2015). Toll-like receptor 5 in obesity: The role of gut microbiota and adipose tissue inflammation. Obesity.

[109] Uchida M., Oyanagi E., Kawanishi N., Iemitsu M., Miyachi M., Kremenik M.J., Onodera S., Yano H. (2014). Exhaustive exercise increases the TNF-α production in response to flagellin via the upregulation of toll-like receptor 5 in the large intestine in mice. Immunol. Lett..

[110] Al-Daghri N.M., Clerici M., Al-Attas O., Forni D., Alokail M.S., Alkharfy K.M., Sabico S., Mohammed A.K., Cagliani R., Sironi M. (2013). A Nonsense Polymorphism (R392X) in TLR5 Protects from Obesity but Predisposes to Diabetes. J. Immunol..

[111] Kim D., Go H.S., Jeon E.J., Nguyen T.Q.T., Kim D.Y., Park H., Eom H., Kim S.Y., Park S.C., Cho K.A. (2025). The Impact of Toll-Like Receptor 5 on Liver Function in Age-Related Metabolic Disorders. Aging Cell.

[112] Pearson J.A., Hu Y., Peng J., Wong F.S., Wen L. (2024). TLR5-deficiency controls dendritic cell subset development in an autoimmune diabetes-susceptible model. Front. Immunol..

[113] Wu S.-C., Rau C.-S., Lu T.-H., Tzeng S.-L., Wu Y.-C., Wu C.-J., Lin C.-W., Hsieh C.-H., Cantarini L. (2015). Effect of Weight-Reduction in Obese Mice Lacking Toll-Like Receptor 5 and C57BL/6 Mice Fed a Low-Fat Diet. Mediat. Inflamm..

[114] Mosquera M.J., Kim S., Zhou H., Jing T.T., Luna M., Guss J.D., Reddy P., Lai K., Leifer C.A., Singh A. (2019). Immunomodulatory nanogels overcome restricted immunity in a murine model of gut microbiome-mediated metabolic syndrome. Sci. Adv..

[115] Vijay-Kumar M., Aitken J.D., Carvalho F.A., Cullender T.C., Mwangi S., Srinivasan S., Sitaraman S.V., Knight R., Ley R.E., Gewirtz A.T. (2010). Metabolic syndrome and altered gut microbiota in mice lacking toll-like receptor 5. Science.

[116] Letran S.E., Lee S.-J., Atif S.M., Flores-Langarica A., Uematsu S., Akira S., Cunningham A.F., McSorley S.J. (2011). TLR5-Deficient Mice Lack Basal Inflammatory and Metabolic Defects but Exhibit Impaired CD4 T Cell Responses to a Flagellated Pathogen. J. Immunol..

[117] Zhang W., Hartmann R., Tun H.M., Elson C.O., Khafipour E., Garvey W.T., Claret M. (2016). Deletion of the toll-like receptor 5 gene per se does not determine the gut microbiome profile that induces metabolic syndrome: Environment trumps genotype. PLoS ONE.

[118] Hamieh A., Mallaret G., Meleine M., Lashermes A., Roumeau S., Boudieu L., Barbier J., Aissouni Y., Ardid D., Gewirtz A. (2021). Toll-like receptor 5 knock-out mice exhibit a specific low level of anxiety. Brain Behav. Immun..

[119] Arias-Loste M.T., Iruzubieta P., Puente Á., Ramos D., Cruz C.S., Estébanez Á., Llerena S., Alonso-Martín C., Segundo D.S., Álvarez L. (2016). Increased expression profile and functionality of TLR6 in peripheral blood mononuclear cells and hepatocytes of morbidly obese patients with non-alcoholic fatty liver disease. Int. J. Mol. Sci..

[120] Kakalij R.M., Dsouza D.L., Boesen E.I. (2022). Development of High Fat Diet-Induced Hyperinsulinemia in Mice Is Enhanced by Co-treatment With a TLR7 Agonist. Front. Physiol..

[121] Kakalij R.M., Dsouza D.L., Ha L., Boesen E.I. (2024). TLR7 activation by imiquimod worsens glycemic control in female FVB/N mice consuming a high-fat diet. Physiol. Rep..

[122] Alshammari T.K., Alghamdi H., Green T.A., Niazy A., Alkahdar L., Alrasheed N., Alhosaini K., Alswayyed M., Elango R., Laezza F. (2019). Assessing the role of toll-like receptor in isolated, standard and enriched housing conditions. PLoS ONE.

[123] Hanna Kazazian N., Wang Y., Roussel-Queval A., Marcadet L., Chasson L., Laprie C., Desnues B., Charaix J., Irla M., Alexopoulou L. (2019). Lupus Autoimmunity and Metabolic Parameters Are Exacerbated Upon High Fat Diet-Induced Obesity Due to TLR7 Signaling. Front. Immunol..

[124] Kubo Y., Yanagawa Y., Matsumoto M., Hiraide S., Kobayashi M., Togashi H. (2012). Toll-like receptor 7-mediated enhancement of contextual fear memory in mice. Pharmacol. Biochem. Behav..

[125] Deng L., Gao R., Chen H., Jiao B., Zhang C., Wei L., Yan C., Ye-Lehmann S., Zhu T., Chen C. (2024). Let-7b-TLR7 Signaling Axis Contributes to the Anesthesia/Surgery-Induced Cognitive Impairment. Mol. Neurobiol..

[126] Hung Y.-F., Hsueh Y.-P. (2021). TLR7 and IL-6 differentially regulate the effects of rotarod exercise on the transcriptomic profile and neurogenesis to influence anxiety and memory. iScience.

[127] Hung Y.-F., Chen C.-Y., Li W.-C., Wang T.-F., Hsueh Y.-P. (2018). Tlr7 deletion alters expression profiles of genes related to neural function and regulates mouse behaviors and contextual memory. Brain Behav. Immun..

[128] Sheng J.A., Christenson J.R., Schwerdtfeger L.A., Tobet S.A. (2024). Maternal immune activation by toll-like receptor 7 agonist during mid-gestation increases susceptibility to blood-brain barrier leakage after puberty. Brain Behav. Immun. Integr..

[129] Sheng J.A., Tobet S.A. (2024). Maternal immune activation with toll-like receptor 7 agonist during mid-gestation alters juvenile and adult developmental milestones and behavior. J. Neuroendocr..

[130] Balan I., Patterson R., Boero G., Krohn H., O’BUckley T.K., Meltzer-Brody S., Morrow A.L. (2023). Brexanolone therapeutics in post-partum depression involves inhibition of systemic inflammatory pathways. EBioMedicine.

[131] Shintani Y., Kapoor A., Kaneko M., Smolenski R., D’Acquisto F., Coppen S.R., Harada-Shoji N., Lee H.J., Thiemermann C., Takashima S. (2013). TLR9 mediates cellular protection by modulating energy metabolism in cardiomyocytes and neurons. Proc. Natl. Acad. Sci. USA.

[132] Liu Y., Nguyen P.T., Wang X., Zhao Y., Meacham C.E., Zou Z., Bordieanu B., Johanns M., Vertommen D., Wijshake T. (2020). TLR9 and beclin 1 crosstalk regulates muscle AMPK activation in exercise. Nature.

[133] Zhao Y., Pu D., Sun Y., Chen J., Luo C., Wang M., Zhou J., Lv A., Zhu S., Liao Z. (2018). High glucose-induced defective thrombospondin-1 release from astrocytes via TLR9 activation contributes to the synaptic protein loss. Exp. Cell Res..

[134] Scholtzova H., Do E., Dhakal S., Sun Y., Liu S., Mehta P.D., Wisniewski T. (2017). Innate Immunity Stimulation via Toll-Like Receptor 9 Ameliorates Vascular Amyloid Pathology in Tg-SwDI Mice with Associated Cognitive Benefits. J. Neurosci..

[135] Tauber S.C., Ebert S., Weishaupt J.H., Reich A., Nau R., Gerber J. (2009). Stimulation of toll-like receptor 9 by chronic intraventricular unmethylated cytosine-guanine DNA infusion causes neuroinflammation and impaired spatial memory. J. Neuropathol. Exp. Neurol..

[136] Jovasevic V., Wood E.M., Cicvaric A., Zhang H., Petrovic Z., Carboncino A., Parker K.K., Bassett T.E., Moltesen M., Yamawaki N. (2024). Formation of memory assemblies through the DNA-sensing TLR9 pathway. Nature.

[137] Zimmerman G., Shaltiel G., Barbash S., Cohen J., Gasho C.J., Shenhar-Tsarfaty S., Shalev H., Berliner S.A., Shelef I., Shoham S. (2012). Post-traumatic anxiety associates with failure of the innate immune receptor TLR9 to evade the pro-inflammatory NFκB pathway. Transl. Psychiatry.

[138] Wu H., Bao H., Liu C., Zhang Q., Huang A., Quan M., Li C., Xiong Y., Chen G., Hou L. (2022). Extracellular Nucleosomes Accelerate Microglial Inflammation via C-Type Lectin Receptor 2D and Toll-Like Receptor 9 in mPFC of Mice With Chronic Stress. Front. Immunol..

[139] Tripathi A., Bartosh A., Whitehead C., Pillai A. (2023). Activation of cell-free mtDNA-TLR9 signaling mediates chronic stress-induced social behavior deficits. Mol. Psychiatry.

[140] Li H., Zhao J., Chen M., Tan Y., Yang X., Caudle Y., Yin D. (2013). Toll-like receptor 9 is required for chronic stress-induced immune suppression. Neuroimmunomodulation.

[141] Sindhu S., Akhter N., Kochumon S., Thomas R., Wilson A., Shenouda S., Tuomilehto J., Ahmad R. (2018). Increased expression of the innate immune receptor TLR10 in obesity and Type-2 diabetes: Association with ROS-mediated oxidative stress. Cell. Physiol. Biochem..

[142] Boutens L., Mirea A.-M., Munckhof I.v.D., Doppenberg-Oosting M., Jaeger M., Hijmans A., Netea M.G., Joosten L.A., Stienstra R. (2018). A role for TLR10 in obesity and adipose tissue morphology. Cytokine.

[143] Kim S.-J., Choi Y., Choi Y.-H., Park T. (2012). Obesity activates toll-like receptor-mediated proinflammatory signaling cascades in the adipose tissue of mice. J. Nutr. Biochem..

[144] Song C., Lv W., Li Y., Nie P., Lu J., Geng Y., Heng Z., Song L. (2021). Alleviating the effect of quinoa and the underlying mechanism on hepatic steatosis in high-fat diet-fed rats. Nutr. Metab..

[145] Sharma I., Priya I., Sharma S., Gupta S., Arora M., Mahajan R., Kapoor N. (2022). Association of toll-like receptor 2 gene polymorphism (rs3804099) with susceptibility to Schizophrenia risk in the Dogra population of Jammu region, North India. Eur. J. Psychiatry.

[146] Dunstan I.K., McLeod R., Radford-Smith D.E., Xiong W., Pate T., Probert F., Anthony D.C. (2024). Unique pathways downstream of TLR-4 and TLR-7 activation: Sex-dependent behavioural, cytokine, and metabolic consequences. Front. Cell. Neurosci..

[147] Smith A.K., Conneely K.N., Kilaru V., Mercer K.B., Weiss T.E., Bradley B., Tang Y., Gillespie C.F., Cubells J.F., Ressler K.J. (2011). Differential Immune System DNA Methylation and Cytokine Regulation in Post-Traumatic Stress Disorder. Am. J. Med. Genet. Part B Neuropsychiatr. Genet..

[148] Hossack M.R., Reid M.W., Aden J.K., Gibbons T., Noe J.C., Willis A.M. (2020). Adverse Childhood Experience, Genes, and PTSD Risk in Soldiers: A Methylation Study. Mil. Med..

[149] Liu B., Huang D., Guo Y., Sun X., Chen C., Zhai X., Jin X., Zhu H., Li P., Yu W. (2022). Recent advances and perspectives of postoperative neurological disorders in the elderly surgical patients. CNS Neurosci. Ther..

[150] Evered L., Silbert B., Knopman D.S., Scott D.A., DeKosky S.T., Rasmussen L.S., Oh E.S., Crosby G., Berger M., Eckenhoff R.G. (2018). Recommendations for the nomenclature of cognitive change associated with Anaesthesia and surgery—2018. Anesthesiology.

[151] Safavynia S.A., Goldstein P.A. (2019). The role of neuroinflammation in postoperative cognitive dysfunction: Moving from hypothesis to treatment. Front. Psychiatry.

[152] Femenía T., Giménez-Cassina A., Codeluppi S., Fernández-Zafra T., Katsu-Jiménez Y., Terrando N., Eriksson L.I., Gómez-Galán M. (2018). Disrupted neuroglial metabolic coupling after peripheral surgery. J. Neurosci..

[153] Yao Y., Lin D., Chen Y., Liu L., Wu Y., Zheng X. (2023). Fluoxetine alleviates postoperative cognitive dysfunction by attenuating TLR4/MyD88/NF-κB signaling pathway activation in aged mice. Inflamm. Res..

[154] Lim J.S., Jeon E.J., Go H.S., Kim H.-J., Kim K.Y., Nguyen T.Q.T., Lee D.Y., Kim K.S., Pietrocola F., Hong S.H. (2024). Mucosal TLR5 activation controls healthspan and longevity. Nat. Commun..

[155] Ma Y., Li J., Chiu I., Wang Y., Sloane J.A., Lü J., Kosaras B., Sidman R.L., Volpe J.J., Vartanian T. (2006). Toll-like receptor 8 functions as a negative regulator of neurite outgrowth and inducer of neuronal apoptosis. J. Cell Biol..

[156] Seizer L., Rahimi S., Santos-Sierra S., Drexel M., Biagini G. (2022). Expression of toll like receptor 8 (TLR8) in specific groups of mouse hippocampal interneurons. PLoS ONE.

[157] Patel A.G., Nehete P.N., Krivoshik S.R., Pei X., Cho E.L., Nehete B.P., Ramani M.D., Shao Y., Williams L.E., Wisniewski T. (2021). Innate immunity stimulation via CpG oligodeoxynucleotides ameliorates Alzheimer’s disease pathology in aged squirrel monkeys. Brain.

[158] Atarashi N., Morishita M., Matsuda S. (2024). Activation of innate immune receptor TLR9 by mitochondrial DNA plays essential roles in the chemical long-term depression of hippocampal neurons. J. Biol. Chem..

[159] Khariv V., Pang K., Servatius R.J., David B.T., Goodus M.T., Beck K.D., Heary R.F., Elkabes S. (2013). Toll-like receptor 9 deficiency impacts sensory and motor behaviors. Brain Behav. Immun..

[160] Patel V., Patel A., McArdle J. (2016). Synaptic abnormalities of mice lacking toll-like receptor (TLR)-9. Neuroscience.

[161] Capuron L., Miller A.H. (2011). Immune system to brain signaling: Neuropsychopharmacological implications. Pharmacol. Ther..

[162] Hotamisligil G.S. (2006). Inflammation and metabolic disorders. Nature.

[163] Dasu M.R., Devaraj S., Park S., Jialal I. (2010). Increased toll-like receptor (TLR) activation and TLR ligands in recently diagnosed type 2 diabetic subjects. Diabetes Care.

[164] Scholtes V.P., Versteeg D., de Vries J.-P.P., Hoefer I.E., Schoneveld A.H., Stella P.R., Doevendans P.A., van Keulen K.J., de Kleijn D.P., Moll F.L. (2011). Toll-like receptor 2 and 4 stimulation elicits an enhanced inflammatory response in human obese patients with atherosclerosis. Clin. Sci..

[165] Ahmad R., Al-Mass A., Atizado V., Al-Hubail A., Al-Ghimlas F., Al-Arouj M., Bennakhi A., Dermime S., Behbehani K. (2012). Elevated expression of the toll like receptors 2 and 4 in obese individuals: Its significance for obesity-induced inflammation. J. Inflamm..

[166] Shechter R., London A., Kuperman Y., Ronen A., Rolls A., Chen A., Schwartz M. (2013). Hypothalamic neuronal toll-like receptor 2 protects against age-induced obesity. Sci. Rep..

[167] Xu H., Tian X., Wang Y., Lin J., Zhu B., Zhao C., Wang B., Zhang X., Sun Y., Li N. (2024). Exercise Promotes Hippocampal Neurogenesis in T2DM Mice via Irisin/TLR4/MyD88/NF-κB-Mediated Neuroinflammation Pathway. Biology.

[168] Thomalla M., Schmid A., Hehner J., Koehler S., Neumann E., Müller-Ladner U., Schäffler A., Karrasch T. (2022). Toll-like Receptor 7 (TLR7) Is Expressed in Adipocytes and the Pharmacological TLR7 Agonist Imiquimod and Adipocyte-Derived Cell-Free Nucleic Acids (cfDNA) Regulate Adipocyte Function. Int. J. Mol. Sci..

[169] Li Q., Yan Y., Liu J., Huang X., Zhang X., Kirschning C., Xu H.C., Lang P.A., Dittmer U., Zhang E. (2019). Toll-Like Receptor 7 Activation Enhances CD8+ T Cell Effector Functions by Promoting Cellular Glycolysis. Front. Immunol..

[170] Yano H., Uchida M., Nakai R., Ishida K., Kato Y., Kawanishi N., Shiva D. (2010). Exhaustive exercise reduces TNF-α and IFN-α production in response to R-848 via toll-like receptor 7 in mice. Eur. J. Appl. Physiol..

[171] Kochumon S., Al Madhoun A., Al-Rashed F., Thomas R., Sindhu S., Al-Ozairi E., Al-Mulla F., Ahmad R. (2020). Elevated adipose tissue associated IL-2 expression in obesity correlates with metabolic inflammation and insulin resistance. Sci. Rep..

[172] Shang W., Xu R., Xu T., Wu M., Xu J., Wang F. (2020). Ovarian Cancer Cells Promote Glycolysis Metabolism and TLR8-Mediated Metabolic Control of Human CD4^+^T Cells. Front. Oncol..

[173] Li L., Liu X., Sanders K.L., Edwards J.L., Ye J., Si F., Gao A., Huang L., Hsueh E.C., Ford D.A. (2019). TLR8-Mediated Metabolic Control of Human Treg Function: A Mechanistic Target for Cancer Immunotherapy. Cell Metab..

[174] Michalek R.D., Gerriets V.A., Jacobs S.R., Macintyre A.N., MacIver N.J., Mason E.F., Sullivan S.A., Nichols A.G., Rathmell J.C. (2011). Cutting Edge: Distinct Glycolytic and Lipid Oxidative Metabolic Programs Are Essential for Effector and Regulatory CD4+ T Cell Subsets. J. Immunol..

[175] Chong H., Wei Z., Na M., Sun G., Zheng S., Zhu X., Xue Y., Zhou Q., Guo S., Xu J. (2020). The PGC-1α/NRF1/miR-378a axis protects vascular smooth muscle cells from FFA-induced proliferation, migration and inflammation in atherosclerosis. Atherosclerosis.

[176] Banerjee A., Chatterji U. (2024). Prevalence of perturbed gut microbiota in pathophysiology of arsenic-induced anxiety- and depression-like behaviour in mice. Chemosphere.

[177] Zhang K., Chen L., Yang J., Liu J., Li J., Liu Y., Li X., Chen L., Hsu C., Zeng J. (2023). Gut microbiota-derived short-chain fatty acids ameliorate methamphetamine-induced depression- and anxiety-like behaviors in a Sigmar-1 receptor-dependent manner. Acta Pharm. Sin. B.

[178] Wang Y., Miao F., Wang J., Zheng M., Yu F., Yi Y. (2024). The Ameliorative and Neuroprotective Effects of Dietary Fibre on Hyperuricaemia Mice: A Perspective from Microbiome and Metabolome. Br. J. Nutr..

[179] Wei H., Yu C., Zhang C., Ren Y., Guo L., Wang T., Chen F., Li Y., Zhang X., Wang H. (2023). Butyrate ameliorates chronic alcoholic central nervous damage by suppressing microglia-mediated neuroinflammation and modulating the microbiome-gut-brain axis. Biomed. Pharmacother..

[180] Murray E., Sharma R., Smith K.B., Mar K.D., Barve R., Lukasik M., Pirwani A.F., Malette-Guyon E., Lamba S., Thomas B.J. (2019). Probiotic consumption during puberty mitigates LPS-induced immune responses and protects against stress-induced depression- and anxiety-like behaviors in adulthood in a sex-specific manner. Brain Behav. Immun..

[181] Fields C.T., Chassaing B., Castillo-Ruiz A., Osan R., Gewirtz A.T., de Vries G.J. (2018). Effects of gut-derived endotoxin on anxiety-like and repetitive behaviors in male and female mice. Biol. Sex Differ..

[182] Zamani M., Alizadeh-Tabari S., Zamani V. (2019). Systematic review with meta-analysis: The prevalence of anxiety and depression in patients with irritable bowel syndrome. Aliment. Pharmacol. Ther..

[183] Clarke G., McKernan D.P., Gaszner G., Quigley E.M., Cryan J.F., Dinan T.G. (2012). A distinct profile of tryptophan metabolism along the kynurenine pathway downstream of toll-like receptor activation in irritable bowel syndrome. Front. Pharmacol..

[184] McKernan D.P., Gaszner G., Quigley E.M., Cryan J.F., Dinan T.G. (2011). Altered peripheral toll-like receptor responses in the irritable bowel syndrome. Aliment. Pharmacol. Ther..

